# Transcriptomic and biochemical analysis of upland cotton (*Gossypium hirsutum*) and a chromosome segment substitution line from *G. hirsutum* × *G. barbadense* in response to *Verticillium dahliae* infection

**DOI:** 10.1186/s12870-018-1619-4

**Published:** 2019-01-11

**Authors:** Peng-tao Li, Md. Harun or Rashid, Ting-ting Chen, Quan-wei Lu, Qun Ge, Wan-kui Gong, Ai-ying Liu, Ju-wu Gong, Hai-hong Shang, Xiao-ying Deng, Jun-wen Li, Shao-qi Li, Xiang-hui Xiao, Rui-xian Liu, Qi Zhang, Li Duan, Xian-yan Zou, Zhen Zhang, Xiao Jiang, Ya Zhang, Ren-hai Peng, Yu-zhen Shi, You-lu Yuan

**Affiliations:** 1 State Key Laboratory of Cotton Biology, Key Laboratory of Biological and Genetic Breeding of Cotton, The Ministry of Agriculture, Institute of Cotton Research, Chinese Academy of Agricultural Science, Anyang, 455000 Henan China; 2 School of Biotechnology and Food Engineering, Anyang Institute of Technology, Anyang, 455000 Henan China; 3 College of Agriculture, South China Agricultural University, Guangzhou, 510642 Guangdong China

**Keywords:** *Gossypium hirsutum*, Chromosome segment substitution lines, Verticillium wilt, Biochemical tests, Transcriptome analysis

## Abstract

**Background:**

Verticillium wilt (VW), also known as “cotton cancer,” is one of the most destructive diseases in global cotton production that seriously impacts fiber yield and quality. Despite numerous attempts, little significant progress has been made in improving the VW resistance of upland cotton. The development of chromosome segment substitution lines (CSSLs) from *Gossypium hirsutum* × *G. barbadense* has emerged as a means of simultaneously developing new cotton varieties with high-yield, superior fiber, and resistance to VW.

**Results:**

In this study, VW-resistant investigations were first conducted in an artificial greenhouse, a natural field, and diseased nursery conditions, resulting in the identification of one stably VW-resistant CSSL, MBI8255, and one VW-susceptible *G. hirsutum*, CCRI36, which were subsequently subjected to biochemical tests and transcriptome sequencing during V991 infection (0, 1, and 2 days after inoculation). Eighteen root samples with three replications were collected to perform multiple comparisons of enzyme activity and biochemical substance contents. The findings indicated that VW resistance was positively correlated with peroxidase and polyphenol oxidase activity, but negatively correlated with malondialdehyde content. Additionally, RNA sequencing was used for the same root samples, resulting in a total of 77,412 genes, of which 23,180 differentially expressed genes were identified from multiple comparisons between samples. After Gene Ontology and Kyoto Encyclopedia of Genes and Genomes enrichment analysis on the expression profiles identified using Short Time-series Expression Miner, we found that the metabolic process in the biological process, as well as the pathways of phenylpropanoid biosynthesis and plant hormone signal transduction, participated significantly in the response to VW. Gene functional annotation and expression quantity analysis indicated the important roles of the phenylpropanoid metabolic pathway and oxidation-reduction process in response to VW, which also provided plenty of candidate genes related to plant resistance.

**Conclusions:**

This study concentrates on the preliminary response to V991 infection by comparing the VW-resistant CSSL and its VW-susceptible recurrent parent. Not only do our findings facilitate the culturing of new resistant varieties with high yield and superior performance, but they also broaden our understanding of the mechanisms of cotton resistance to VW.

**Electronic supplementary material:**

The online version of this article (10.1186/s12870-018-1619-4) contains supplementary material, which is available to authorized users.

## Background

Cotton, as one of the most important commercial crops, is the most widely cultivated fiber plant globally, and it is of great economic and social significance [1]. Despite being comprised of 46 diploid (2n = 2x = 26) and 5 allotetraploid (2n = 4x = 52) species, only 4 *Gossypium* species are widely cultivated, namely, *G. arboreum*, *G. herbaceum*, *G. hirsutum,* and *G. barbadense* [2]*.* As the major allotetraploid cotton species, upland cotton (*G. hirsutum*) and Sea Island cotton (*G. barbadense*) contribute more than 95% fiber capacity, and they originated from a hybridization event between *G. arboreum* (A_t_ sub-genome) and *G. raimondii* (D_t_ sub-genome) 1–2 million years ago [3]. Verticillium wilt (VW), a representative vascular disease mainly caused by the infection of *Verticillium dahliae* Kleb., and the soil-borne fungus can cause leaf yellowing, wilt, defoliation, and even death [4]. Cotton VW is the most destructive disease, presently tremendously affecting cotton yield and fiber performance [5, 6].

Initially described on upland cotton in 1914 in the USA, VW was subsequently introduced into China via the importation of American cotton species, resulting in substantial cotton yield losses [7]. *Verticillium dahliae* can cause wilt disease on more than 200 plant species, indicating its extensive range of host plants [8, 9]. The microsclerotia (melanized survival structures) formed by the soil-borne pathogen can survive in the soil for over 10 years, with germination being triggered by root exudate signals [10, 11]. Unfortunately, there are no appropriate commercial fungicides available for *G. hirsutum*, while *G. barbadense,* despite possessing innate resistance to VW and premium fiber quality, experiences low-fiber productivity upon infection [12]. The above-mentioned conditions reduce the effectiveness of common cultivation practices, such as crop rotation, chemical fumigation, and soil conditioning [13]. Thus, the development of new cotton cultivars possessing VW resistance by means of traditional breeding and transgenic strategies is the most practical and cost effective method to manage cotton VW [14–16]. Given the increasing human population and diminishing arable land, the development of novel varieties harboring high yield, superior fiber quality, and VW resistance remains a significant topic in cotton breeding. In response, chromosome segment substitution lines (CSSLs) have emerged as a means of combining the advantages of both upland and Sea Island cotton. Through conventional breeding methods, such as hybridization, backcrossing, selfing, and marker assisted-selection (MAS), CSSLs were developed as the ideal materials for further genome research and crop improvement. CSSLs have been preferentially applied to the quantitative trait locus (QTL) mapping for essential traits, such as yield, quality, disease resistance, and stress tolerance in tomato [17], wheat [18], rice [19, 20], and cotton [21–26].

Plants have evolved two-layered immune mechanisms to protect themselves against various pathogens with different invasion strategies. The first-layer defense principally occurs on the extracellular surface of the host cell, where the pattern recognition receptors (PRRs) detect the pathogen-associated molecular patterns (PAMPs), further causing PAMP-triggered immunity (PTI) by the stimulation of PRRs [27, 28]. Although most pathogens are blocked by the basal immune response, some are able to successfully invade and suppress PTI through the deliverance of pathogenic effector proteins into the host cells via a type III secretion system (TTSS) [29]. Subsequently, the specific resistance (R) genes encoding nucleotide-binding site (NBS) and leucine-rich repeat (LRR) domains recognize these pathogen effectors, ultimately activating the second-layer defense mechanism, namely, effector-triggered immunity (ETI) [27, 28]. Plant defense reactions in response to diverse pathogens are triggered along with the recognition of the PRRs and R genes, further causing a localized activation of programmed cell death (PCD), also known as the hypersensitive response (HR) [30, 31]. Plenty of R proteins in plants share two typical domains: a nucleotide-binding site (NBS) and a C-terminal leucine-rich repeat (LRR) region [32]. The former constitutes part of the nucleotide-binding (NB)-ARC adaptor shared by the apoptotic protease-activating factor 1 (APAF-1), R proteins, and *Caenorhabditis elegans* homolog domain 4 (CED-4) [33], while the latter proteins play a central role in the biological processes of plant growth and development, including the immune response [34]. Based on the N-terminal domain categories, plant NBS-LRR proteins can be classified into toll/interleukin-1 receptors (TIR) and coiled-coil (CC), respectively referred to as TIR-NBS-LRR and CC-NBS-LRR [35, 36].

Along with the rapid development of next-generation sequencing technologies, diploid and allotetraploid *Gossypium* species have been successfully sequenced [37–42], which has improved our understanding of cotton polyploid properties and provided a solid foundation for further functional genomics. Transcriptome sequencing, also known as RNA-Seq, concentrates on gene expression and transcriptional regulation, making it suitable for revealing the molecular mechanisms of particular biological processes. RNA-Seq has been widely applied to identify the key signaling pathways or resistant genes in response to VW not only in cotton [12, 43], but also in *Nicotiana benthamiana* [44], smoke tree [45], tomato [46], sunflower [47], and olive [48]. Furthermore, the phenylalanine pathway, which includes lignin and flavonoid biosynthesis, has been reported to be associated with VW resistance and is similarly regulated by some enzyme activities and biochemical substances, including polyphenol oxidase (PPO), phenylalanine ammonia lyase (PAL), peroxidase (POD), superoxide dismutase (SOD), and malondialdehyde (MAD) [12, 43].

In the present study, VW resistance investigations in artificial greenhouse, natural field, and disease nursery conditions were first conducted, and MBI8255, the VW-resistant CSSL, and CCRI36, the VW-susceptible recurrent parent, were subsequently used to perform biochemical tests during the preliminary stages of the V991 infection process (0, 1, and 2 days after inoculation, DAI), which included assessing the activities of CAT, POD, SOD, PAL, and PPO, and the contents of MDA, proline, soluble sugar, and soluble protein. Additionally, RNA-Seq was carried out on the same samples, and plenty of differentially expressed genes (DEGs) relevant to VW resistance were identified, which were subjected to Gene Ontology (GO) and Kyoto Encyclopedia of Genes and Genomes (KEGG) enrichment analysis. This report not only identified various candidate genes associated with VW resistance, but also identified the key signaling pathways in response to V991 infection, which should elucidate the molecular mechanisms of cotton VW resistance and provide a solid foundation for cotton breeding and genomics research.

## Results

### Phenotypic disease index (DI) in the greenhouse tests

**Fig. 1:**
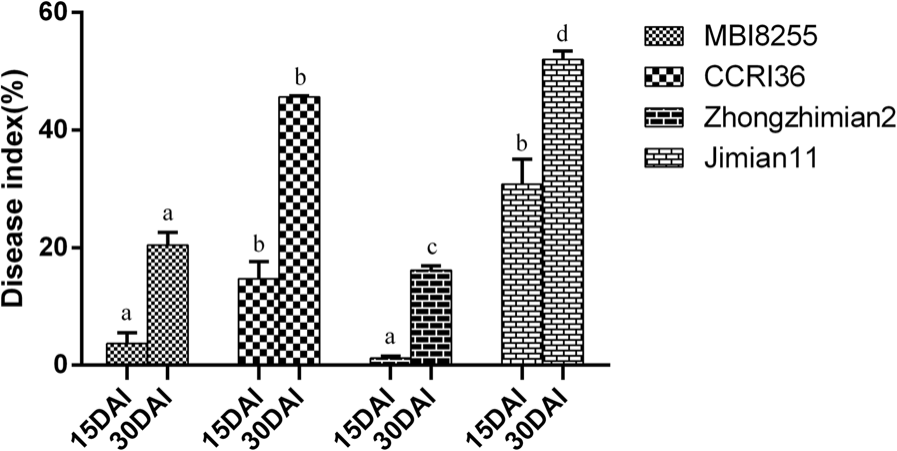
VW DI values of MBI8255, CCRI36 and two controls, the resistant Zhngzhimian2 and the susceptible Jimian11. Data were separately collected at 15 and 30 DAI. The error bars show the standard deviation and a, b, c indicate the significant differences in LSD tests in 15 and 30 DAI, respectively

In order to evaluate the resistance to VW caused by V991, greenhouse tests with three replications of MBI8255, CCRI36, and two controls (Zhongzhimian2 and Jimian11) were conducted in 2015, with the phenotyping characterizations respectively collected at 15 and 30 DAI (Table 1). At 15 DAI, higher DI values were recorded in Jimian11 (27.11%) and CCRI36 (15.38%), which are susceptible to VW, while lower DI values were found in the resistant Zhongzhimian2 (1.10%) and MBI8255 (3.26%). Based on Least Significant Difference (LSD) tests at *P* = 0.05, no significant difference was observed between the DI values of the CSSL MBI8255 and resistant control Zhongzhimian2, which were significantly lower than those of the recurrent parent CCRI36 and the susceptible control Jimian11 (Fig. 1). Furthermore, a similar phenomenon arose in the DI values of the four materials at 30 DAI. The highest DI value was observed in Jimian11 (51.81%), while the lowest DI value was recorded in Zhongzhimian2 (16.18%). The second highest and lowest DI values were separately recorded in CCRI36 (45.67%) and MBI8255 (20.38%). Based on the LSD test, significant differences were found not only between MBI8266 and Zhongzhimian2, but also between CCRI36 and Jimian11, while the DI values of the former two were significantly lower than those of the latter two.

**Table 1:** Descriptive statistics of DI in greenhouse, field and disease nursery tests

Test	Phenotype	Env	MBI8255	CCRI36	Jimian11	Zhongzhimian2
Greenhouse	DI(%)	15DIA	3.26	15.38	27.11	1.1
		30DIA	20.38	45.67	51.81	16.18
Field	DI(%)	AYF15	6.73	27.89	44.74	5.67
		AYM15	23.91	48.16	53.41	17.05
		XJF15	0	5.84	8.7	1.12
		XJM15	29.69	41.59	53.52	27.49
Disease nursery	DI(%)	KFM15	6.82	33.33	56.77

### Phenotypic DI in the natural field and disease nursery tests

To investigate VW resistance in the natural environment, natural field tests of MBI8255, CCRI36, and two controls (Zhongzhimian2 and Jimian11) were respectively performed in Anyang (Henan Province) with one replication and in Shihezi (Xinjiang Province) with one replication in 2015, with the phenotypic identifications being separately carried out at the flowering and maturity stages (Table 1). In the field tests, the DI values of the two controls, Jimian11 and Zhongzhimian2, varied from 44.74 and 5.67% at the flowering stage to 53.41 and 17.05% at the maturity stage in Anyang, while those of MBI8255 and CCRI36 varied from 6.73 and 27.89% at the flowering stage in Anyang to 23.91 and 48.16%, respectively. With regards to the field tests in Xinjiang, the DI values of the VW-resistant Zhongzhimian2 and MBI8255 varied from 1.12% and 0 to 27.49 and 29.69%, and those of the VW-susceptible Jimian11 and CCRI36 varied from 8.7 and 5.84% to 53.52 and 41.59%, respectively.

Disease nursery tests were conducted to evaluate the DI values of MBI8255, CCRI36, and Jimian11 in Kaifeng (Henan Province) with one replication, and only the maturity stage was chosen to determine the phenotypic characteristics, ultimately resulting in DI values of 6.82, 33.33, and 56.77%, respectively.

### Biochemical responses to V991 infection in MBI8255 and CCRI36

**Fig. 2:**
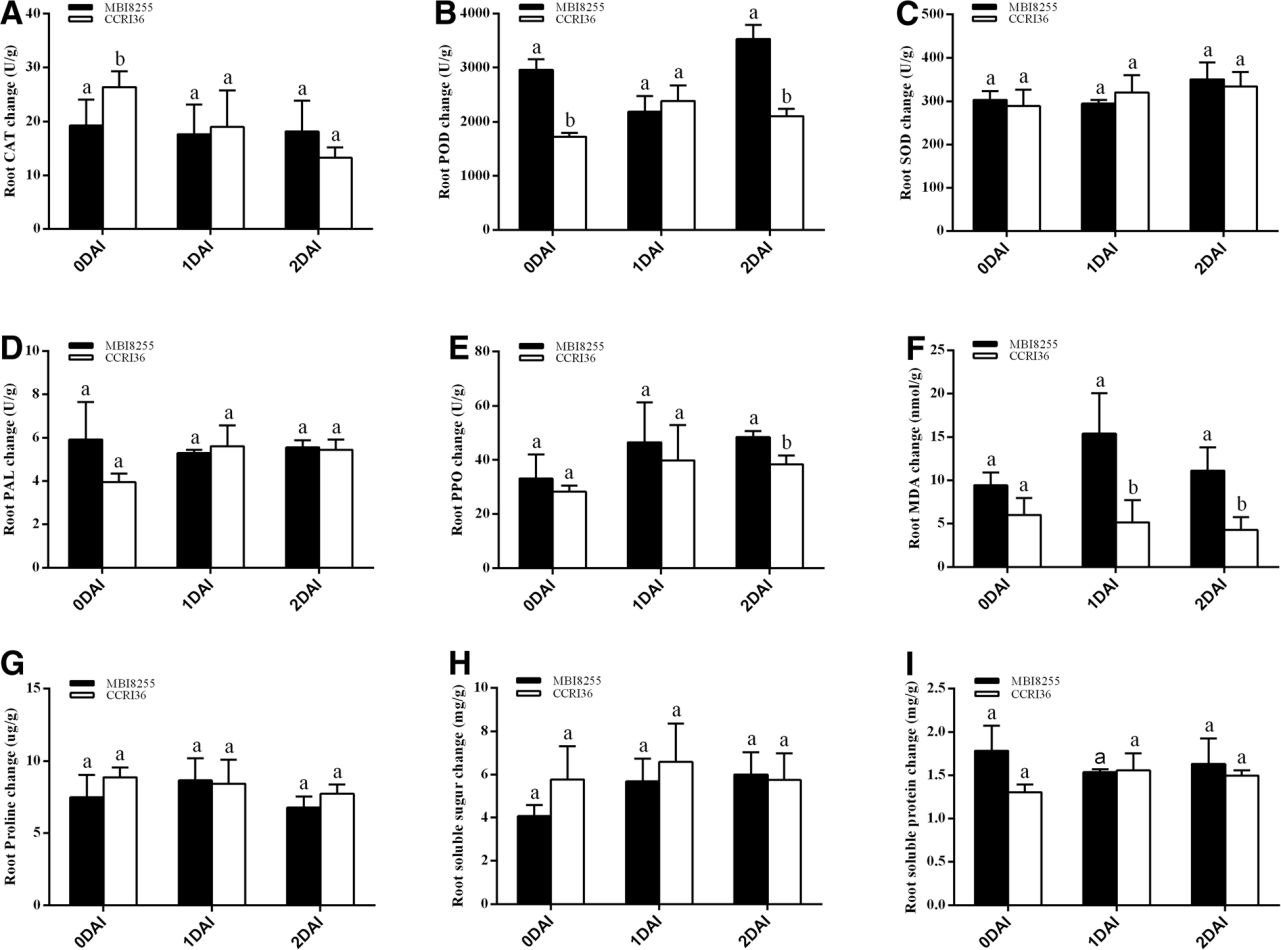
Biochemistry analysis of MBI8255 and CCRI36 in response to V991 infection. **a**-**c**: the activity changes of 3 protective enzymes relevant to VW resistance (CAT, POD and SOD) in root at 0, 1 and 2 DAI infected by V991. **d**-**e**: the activity changes of 2 defensive enzymes relevant to VW resistance (PAL and PPO) in root at 0, 1 and 2 DAI infected by V991. **f**-**i**: the content changes of 4 physiochemical substances relevant to VW resistance (MDA, Proline, soluble sugar and soluble protein) in root at 0, 1 and 2 DAI infected by V991. Three biological replications were performed and the error bars represent the standard deviation

To explore the relationships between cotton VW and the specific signaling pathways or biochemical substances, biochemical tests of MBI8255 and CCRI36 in response to V991 infection were conducted on the enzyme activity or biochemical substances in the roots at 0, 1, and 2 DAI. Three protective enzymes, namely, CAT, POD, and SOD, were chosen to analyze their activity changes during the process of V991 infection. The results are shown in Fig. 2a–c. CAT activity in the roots of CCRI36 gradually decreased from 0 DAI (26.42 U/g) to 2 DAI (13.28 U/g), but exhibited little change in the root samples of MBI8255, in which the highest and lowest activities occurred at 0 DAI (19.24 U/g) and 1 DAI (17.62 U/g). The POD activity first increased from 0 DAI (1728 U/g) to 1 DAI (2389.33 U/g), and then decreased at 2 DAI (2109.33 U/g) in the roots of CCRI36, while an opposite trend was observed in POD activity in the MBI8255 root samples, which first declined from 0 DAI (2957.33 U/g) to 1 DAI (2189.33 U/g) and then peaked at 2 DAI (3528 U/g). In regard to root SOD, its activity in CCRI36 gradually increased from 0 DAI (289.49 U/g) to 2 DAI (334.37 U/g), with the roots of MBI8255 exhibiting the highest and lowest SOD activity at 1 DAI (295.18 U/g) and 2 DAI (350.17 U/g), respectively.

Two defense enzymes relevant to VW resistance, namely, PAL and PPO, were also selected to investigate the activity changes following V991 infection (Fig. 2d–e). Only the PAL and PPO changes in the roots of CCRI36 showed a similar trend, first increasing from 3.94 U/g and 28.32 U/g at 0 DAI to 5.61 U/g and 39.83 U/g at 1 DAI, and then declining to 5.45 U/g and 38.4 U/g at 2 DAI, respectively. The high PAL activity of the roots of MBI8255 at 0 DAI (5.92 U/g) dropped to the lowest value at 1 DAI (5.29 U/g), and then rose to 5.55 U/g at 2 DAI, while the PPO activity in the roots of MBI8255 rose from the lowest value of 33.12 U/g at 0 DAI to the highest value of 48.48 U/g at 2 DAI.

Similarly, the contents of MDA, proline, soluble sugar, and soluble protein were assessed in the roots during V991 infection (Fig. 2f–i). Similar variation patterns were observed in both MDA and proline contents in the roots of the two lines, which showed a gradual decrease in CCRI36, whereas MBI8255 initially rose and then declined. The highest and lowest MDA contents in CCRI36 were 6 nmol/g at 0 DAI and 4.29 nmol/g at 2 DAI, respectively, while those in MBI8255 were 15.43 nmol/g at 1 DAI and 9.43 nmol/g at 0 DAI, respectively. The maximum and minimum proline values in CCRI36 were 8.87 μg/g at 0 DAI and 7.73 μg/g at 2 DAI, respectively, while those in MBI8255 were 8.65 μg/g at 1 DAI and 6.78 μg/g at 2 DAI, respectively. The soluble sugar content of the roots of CCRI36 increased from 5.76 mg/g at 0 DAI to 6.59 mg/g at 1 DAI, and then declined to 5.75 mg/g at 2 DAI. The soluble sugar content in MBI8255 showed a gradual increase from 4.07 mg/g at 0 DAI to 6 mg/g at 2 DAI. The soluble protein content in the roots of CCRI36 first rose from 1.31 mg/g at 0 DAI to 1.55 mg/g at 1 DAI and then declined to 1.50 mg/g at 2 DAI, while MBI8255 showed an opposite pattern, decreasing from 1.79 mg/g at 0 DAI to 1.54 mg/g and then increasing to 1.63 mg/g at 2 DAI.

### Transcriptome sequencing and alignment to the *G. hirsutum* genome

In this study, 18 RNA-Seq libraries were constructed from root samples infected by V991, which were collected at 0, 1, and 2 DAI in MBI8255 and CCRI36 with three biological replications, with the aim of systematically identifying the key genes or pathways affecting cotton resistance to VW. In total, 922.867 million raw reads were obtained, which were filtered for low-quality reads, resulting in 908.547 million clean reads (approximately 136.27 Gb data) with an average of 7.57 G per library (Table 2). Over 91.38% of the Q30 values and no less than 43.44% of the GC content were calculated from the RNA-Seq results, of which the average values were 92.23 and 43.68%, respectively. Subsequently, the clean reads were mapped to the *G. hirsutum* genome using TopHat2 software, and 87.69–91.23% of the clean data were successfully matched to the reference genome, of which 79.13–84.29% and 6.63–8.39% constituted unique and multiple reads, respectively. All of the above-mentioned results implied the reliability of our transcriptome results.

**Table 2:** Throughput and quality of RNA-seq of the 18 libraries

Libraries	Raw reads	Clean reads	Clean bases	Q30 (%)	GC (%)	Total match(%)	Multiple match(%)	Unique match(%)
CCRI36–0I	56,789,392	55,874,790	8.38G	92.32	43.65	90.26	7.16	83.1
CCRI36–0II	54,872,602	54,020,096	8.1G	92.38	43.63	89.86	7.08	82.78
CCRI36–0III	54,595,684	53,962,178	8.09G	92.66	43.8	91.23	7	84.23
CCRI26-1I	52,215,302	51,603,780	7.74G	92.55	43.8	91.65	7.36	84.29
CCRI36–1II	49,878,906	49,240,112	7.39G	92.31	43.84	90.75	7.61	83.15
CCRI36–1III	60,433,302	58,873,730	8.83G	90.45	43.61	87.52	8.39	79.13
CCRI36–2I	55,215,736	54,486,388	8.17G	92.35	43.5	91.02	7.44	83.59
CCRI36–2II	53,009,636	52,206,706	7.83G	91.73	43.99	89.17	6.86	82.3
CCRI36–2III	43,962,200	43,194,444	6.48G	93.13	43.6	87.69	6.63	81.06
MBI8255–0I	46,707,676	45,868,652	6.88G	92.4	43.57	90.9	7.11	83.79
MBI8255–0II	51,016,406	50,290,518	7.54G	92.31	43.68	90.35	7.25	83.1
MBI8255–0III	48,958,146	48,291,880	7.24G	92.27	43.87	90.48	7.16	83.32
MBI8255–1I	47,850,214	47,192,996	7.08G	92.58	43.44	90.71	7.31	83.4
MBI8255–1II	51,331,338	50,606,826	7.59G	92.79	43.64	91.23	8.05	83.19
MBI8255–1III	53,415,870	52,706,364	7.91G	92.84	43.76	90.53	7.17	83.36
MBI8255–2I	47,814,682	46,903,434	7.04G	91.38	43.48	89.53	7.1	82.44
MBI8255–2II	42,917,798	42,231,086	6.33G	91.92	43.61	90.29	7.1	83.19
MBI8255–2III	51,881,984	50,993,450	7.65G	91.74	43.72	89.2	7.15	82.05
Average	51,270,382	50,474,857	7.57G	92.23	43.68	90.13	7.27	82.86

**Fig. 3:**
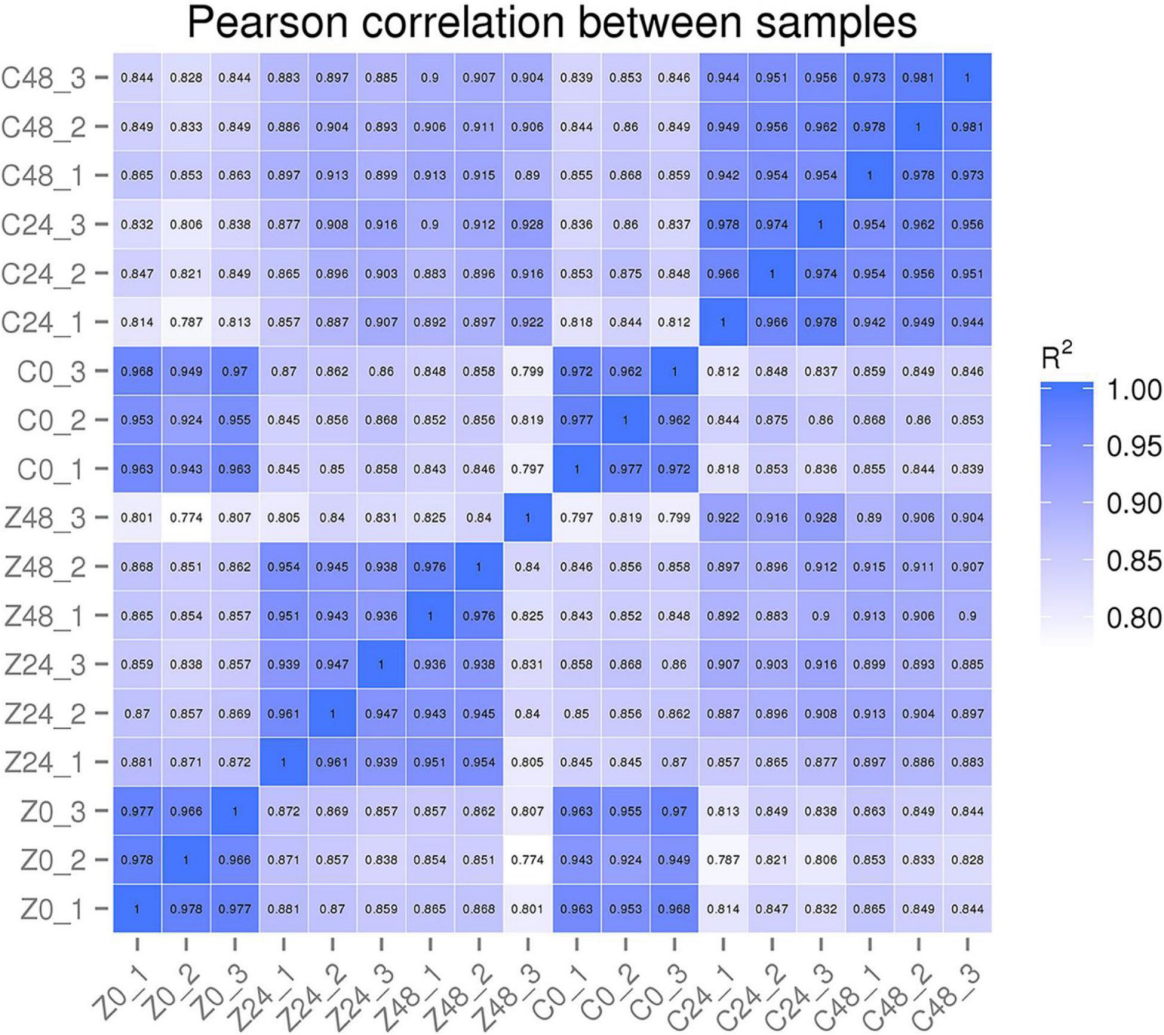
Pearson correlation coefficient analysis of the total genes identified from the 18 samples. Z and C represent CCRI36 and MBI8255, respectively. 0 = 0 DAI,24 = 1 DAI,48 = 2 DAI.

A total of 77,412 genes containing 6934 novel genes were found in our RNA-Seq results, of which the expression quantities were subsequently evaluated based on the Fragments Per Kilobase of transcript per Million mapped reads (FPKM) value. Pearson Correlation Coefficient (PCC) analysis was used to calculate the correlations between the 18 experimental samples, approximately resulting in more than 93.9% similarity in the gene expressions of each sample based on three replications; however, exceptions were observed for 82.5 and 84% of similarities separately identified between CCRI36–2 III and CCRI36–2 I/CCRI36–2 II (Fig. 3). We found 92.4–97% similarity between the gene profiles of CCRI36 and MBI8255 at 0 DAI, whereas the similarities were 85.7–91.6% and 89–91.5%, respectively, at 1 and 2 DAI, which suggested that the two lines at the same stages of V991 infection exhibited a similar gene expression pattern even though the similarities at 1 and 2 DAI were lower than those at 0 DAI. A gradual decrease in expressed gene similarity was observed between CCRI36–0 and CCRI36–1/CCRI36–2, first declining from 0 DAI to 1 DAI and then increasing at 2 DAI in MBI8255. The differences between the two lines might result from the CSSL with distinct *G. barbadense* chromosomal segments.

### Identification of differentially expressed genes (DEGs)

**Fig. 4:**
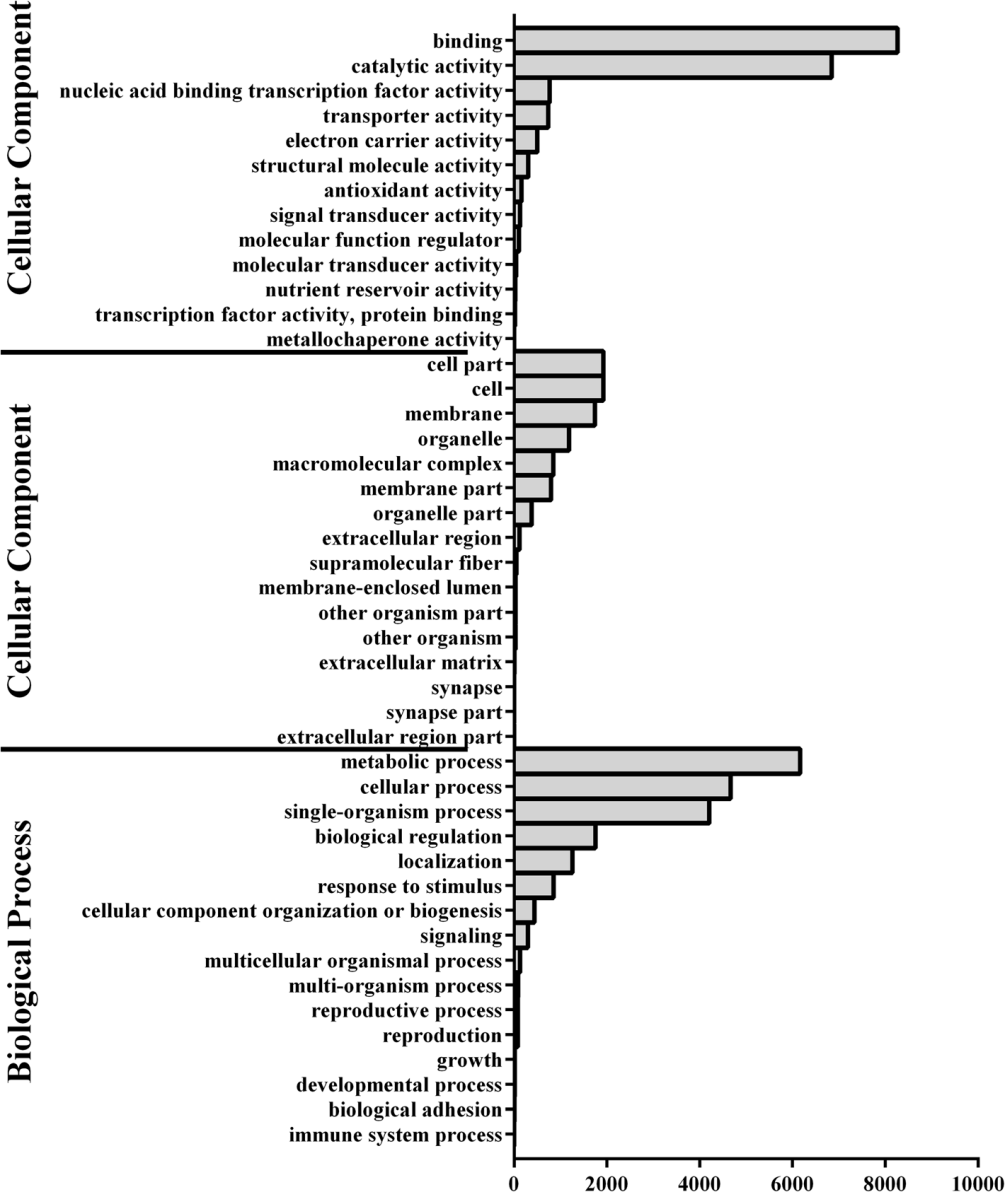
The GO enrichment analysis of the total differentially expressed genes

To investigate the differential responses to V991 infection, three replications per sample in the two lines were merged to conduct pairwise comparisons, and DEGs were identified by DESeq R package (v1.18.0) and were based on the Q-value, with a false discovery rate (FDR)-adjusted cut-off value of < 0.01 and an absolute log_2_Change ≥ 2. A total of 23,180 genes containing 1816 novel genes were differentially expressed in response to V991 infection (Additional file 1). These were subjected to GO enrichment analysis (Fig. 4). Forty-six subcategories were clustered into three categories, specifically biological process, cellular component, and molecular function, and the primary enriched groups in these categories were metabolic process and cellular process, cell and cell part, and binding and catalytic activity.

**Fig. 5:**
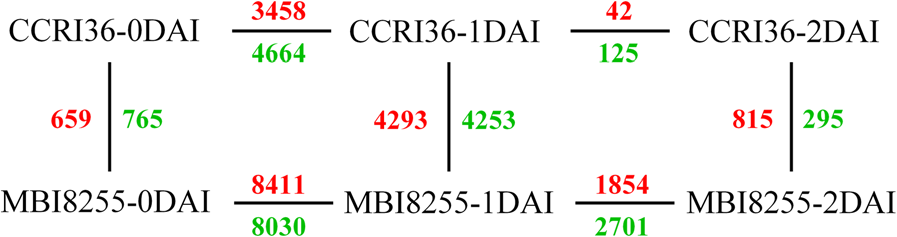
The differentially expressed genes between the different samples. Red and blue numbers represent the up-regulated and down-regulated genes, respectively

**Fig. 6:**
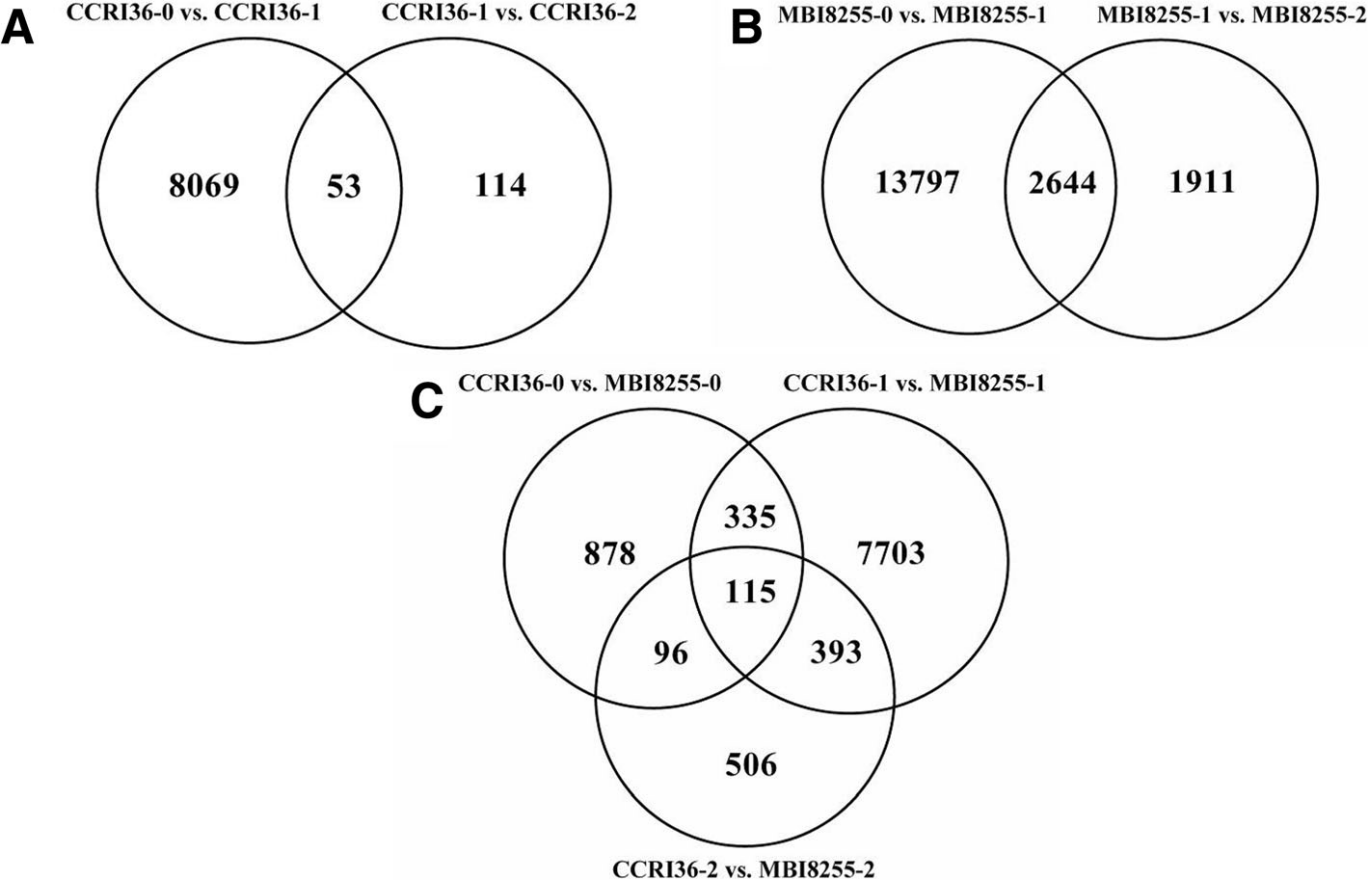
The differentially expressed genes from multiple comparisons between the different samples. **a** represents the comparisons of CCRI36 samples at the different stages, (**b**) represents the comparisons of MBI8255 samples at the different stages, (**c**) represents the comparisons of 2 lines at the same stages

The results of the pairwise comparisons were as follows (Fig. 5): 1424 DEGs from CCRI36–0 vs. MBI8255–0, 8122 DEGs from CCRI36–0 vs. CCRI36–1, 167 DEGs from CCRI36–1 vs. CCRI36–2, 16,441 DEGs from MBI8255–0 vs. MBI8255–1, 4555 DEGs from MBI8255–1 vs. MBI8255–2, 8546 DEGs from MBI8255–1 vs. CCRI36–1, and 1110 DEGs from MBI8255–2 and CCRI36–2. Based on the comparisons between CCRI36–0 vs. CCRI36–1 and CCRI36–1 vs. CCRI36–2 (Fig. 6a), 53 DEGs were commonly identified, while 2644 common DEGs were found in the comparisons of MBI8255–0 vs. MBI8255–1 and MBI8255–1 vs. MBI8255–2 (Fig. 6b). There were 450 and 211 common DEGs identified from the comparisons between CCRI36–0 vs. MBI8255–0 and CCRI36–1 vs. MBI8255–1/CCRI36–2 vs. MBI8255–2 (Fig. 6c), respectively, while 508 common DEGs were observed between CCRI36–1 vs. MBI8255–1 and CCRI36–2 vs. MBI8255–2, ultimately identifying 115 common genes derived from all of the above comparisons.

### Analysis of gene temporal expression patterns

**Fig. 7:**
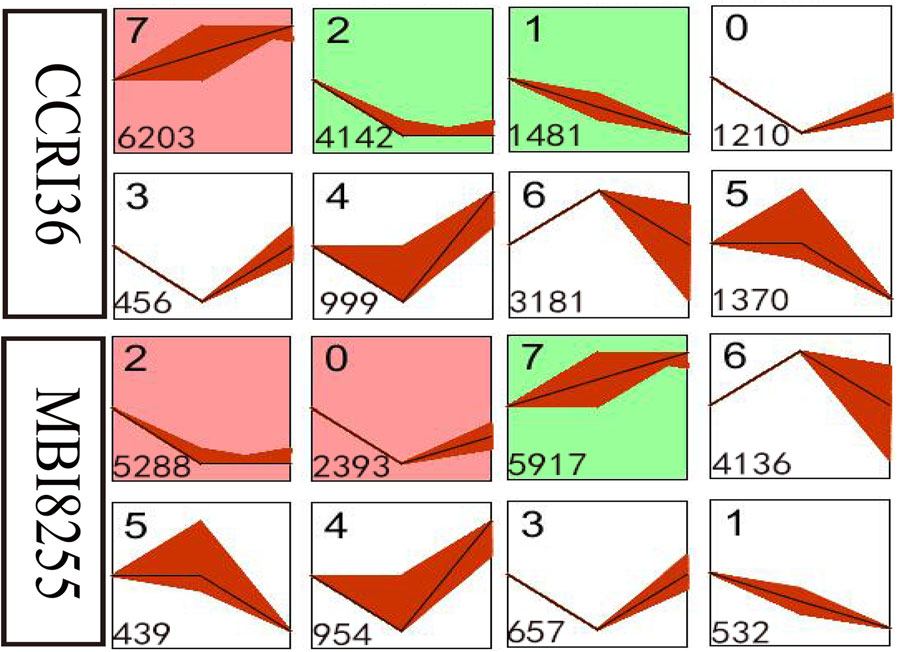
Different gene expression patterns by STEM software in two lines. Each square represents a trend of gene expression. The number in top left corner indicates the profile ID number, and the number in bottom left corner indicates the number of gens in that profile. The clusters and profiles were ordered based on the number of genes and significance (default), respectively

To investigate the temporal patterns of all the 23,180 DEGs, Short Time-series Expression Miner (STEM) analyses were conducted in the two lines. In total, 19,044 DEGs in CCRI36 and 20,316 DEGs in MBI8255 were clustered into eight profiles (Fig. 7), with each profile representing a group of genes with a similar expression pattern (Additional file 2). Among the CCRI36 profiles, 11,827 DEGs (62.1% of 19,044) were further clustered into three profiles (*P*-value ≤0.05), including one up-regulated pattern, namely, profile 7 (6024 DEGs, 31.63%), and two down-regulated patterns, namely, profile 2 (4142 DEGs, 21.75%) and profile 1 (1481 DEGs, 7.78%). Similarly, 13,598 DEGs (66.93% of the 20,316 DEGs) in MBI8255 were clustered into three profiles (*P*-value ≤0.05). Up-regulated profile 7 and down-regulated profile 2 contained 5912 (29.12%) and 5288 (26.03%) DEGs, while profile 0 (2393 DEGs, 11.78%) first decreased and then increased in expression levels.

**Fig. 8:**
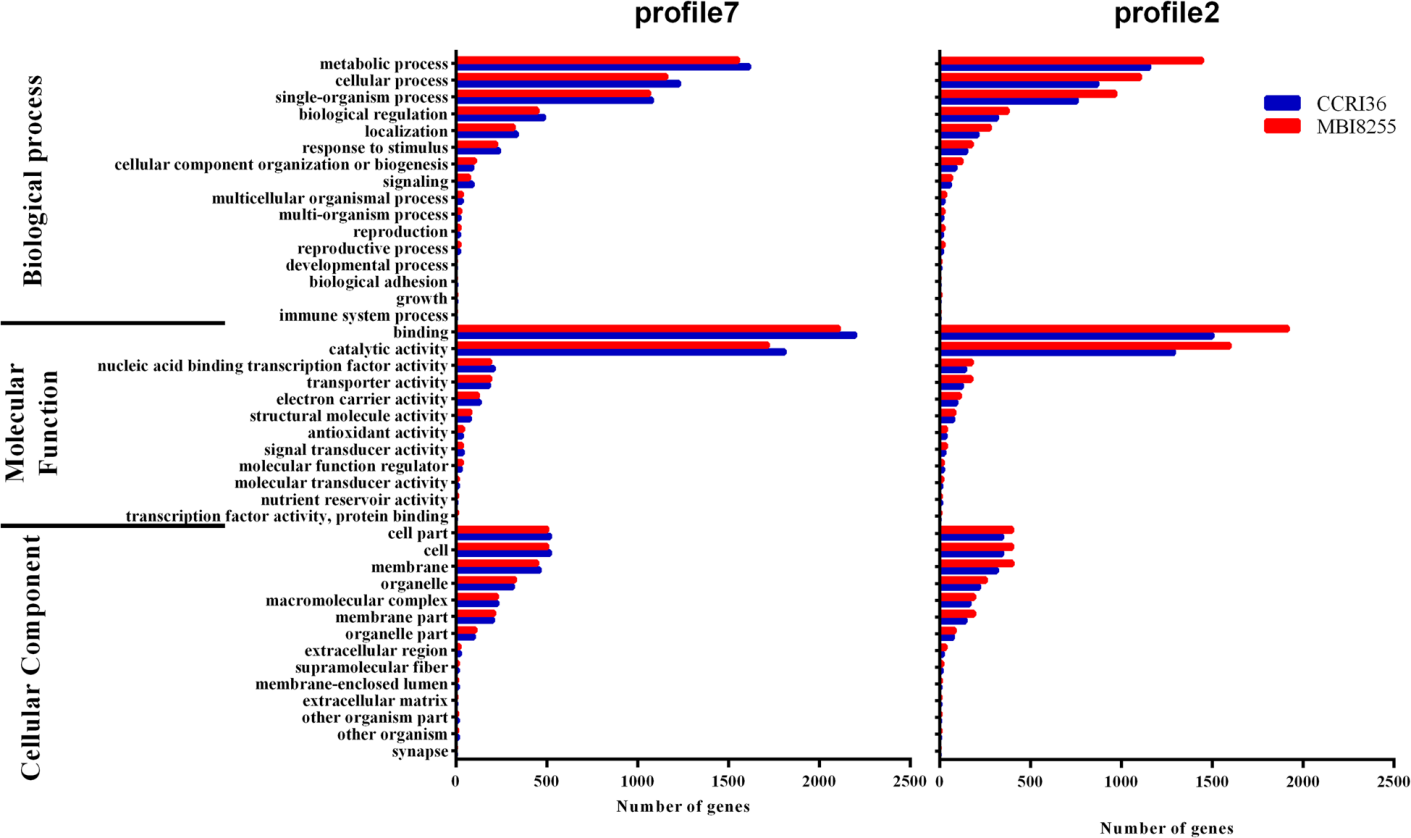
GO classification of profile7 and profile2 in two lines (CCRI36 and MBI8255)

Next, GO enrichment analysis was conducted to identify the putative functional genes from the profiles in the two lines. Profile 7 and profile 2 possessed the most DEGs with significant values (*P*-value ≤0.05) among the eight profiles (Fig. 8). All of the DEGs were classified into three main categories consisting of biological process, molecular function, and cellular component. In combination with the GO term results of the two profiles in CCRI36 and MBI8255, metabolic process and cellular process were the dominant subcategories in biological process, while binding and catalytic activity were the most abundant subcategories in molecular function, and cell part and cell were the top two abundant subcategories in cellular component. In the up-regulated profile 7, more DEGs clustered into the subcategories in CCRI36 than in MBI8255, while on the contrary, a greater number of DEGs clustered into the same subcategories in MBI8255 than in the down-regulated profile 2.

Similarly, the above-mentioned two profiles in CCRI36 and MBI8255 were subjected to KEGG pathway analysis, and the 20 top signaling pathways with the greatest number of DEGs are shown in Table 3. For the up-regulated pattern of profile 7, eight common pathways were significantly enriched in the two lines, namely, phenylpropanoid biosynthesis (ko00940), plant hormone signal transduction (ko04075), circadian rhythm-plant (ko04712), flavonoid biosynthesis (ko00941), stilbenoid, diarylheptanoid and gingerol biosynthesis (ko00945), limonene and pinene degradation (ko00903), galactose metabolism (ko00380), and carotenoid biosynthesis (ko00052). In addition to the above-mentioned pathways, there were only three significantly enriched pathways in CCRI36, including amino sugar and nucleotide sugar metabolism (ko00520), glycolysis/gluconeogenesis (ko00010), and tryptophan metabolism (ko00240). The most enriched pathway in profile 7 of the two lines was similarly annotated to starch and sucrose metabolism (ko00500), while the smallest *P*-value pathways were separately identified as flavonoid biosynthesis in CCRI36 and as phenylpropanoid biosynthesis in MBI8255.

**Table 3:** 20 top KEGG pathways with high representation of the DEGs

Pathway	DEGs with pathway annotation				Pathway ID
	CCRI36 profile7	MBI8255 profile7	CCRI36 profile2	MBI8255 profile2	
Starch and sucrose metabolism	76	72	50	57	ko00500
Phenylpropanoid biosynthesis	73*	71*	49*	56*	ko00940
Plant hormone signal transduction	60*	55*	45*	58*	ko04075
Circadian rhythm - plant	59*	56*	36	48*	ko04712
Flavonoid biosynthesis	52*	42*	25	33*	ko00941
Carbon metabolism	49	38	25	37	ko01200
Stilbenoid, diarylheptanoid and gingerol biosynthesis	47*	46*	26	40*	ko00945
Limonene and pinene degradation	45*	46*	27	39*	ko00903
Ribosome	45	46	32	43	ko03010
Amino sugar and nucleotide sugar metabolism	43*	38	20	28	ko00520
Pentose and glucuronate interconversions	36	31	26	27	ko00040
Biosynthesis of amino acids	32	35	24	31	ko01230
Glycolysis/Gluconeogenesis	28*	23	9	9	ko00010
Protein processing in endoplasmic reticulum	28	20	13	22	ko04141
Pyrimidine metabolism	28	27	24	25	ko00240
Tryptophan metabolism	26*	14	12	11	ko00380
Galactose metabolism	24*	28*	6	10	ko00052
Carotenoid biosynthesis	23*	24*	13	17	ko00906
Plant-pathogen interaction	23	16	13	19	ko04626
Ubiquitin mediated proteolysis	23	16	13	18	ko04120

“*” means the significantly enriched pathways

In the down-regulated profile 2, only two common enriched pathways with significance, namely, phenylpropanoid biosynthesis and plant hormone signal transduction, were observed in the two lines, with the latter pathway indicating not only the smallest *P*-values in both CCRI36 and MBI8255, but also the greatest number of DEGs in MBI8255. Starch and sucrose metabolism was the most enriched pathway in profile 2 of CCRI36. Compared to the pathways in profile 2 of CCRI36, circadian rhythm-plant, flavonoid biosynthesis, stilbenoid, diarylheptanoid and gingerol biosynthesis, and limonene and pinene degradation were the significantly enriched pathways in MBI8255 only.

### Expression profiling of DEGs related to the phenylpropanoid metabolic pathway

**Fig. 9:**
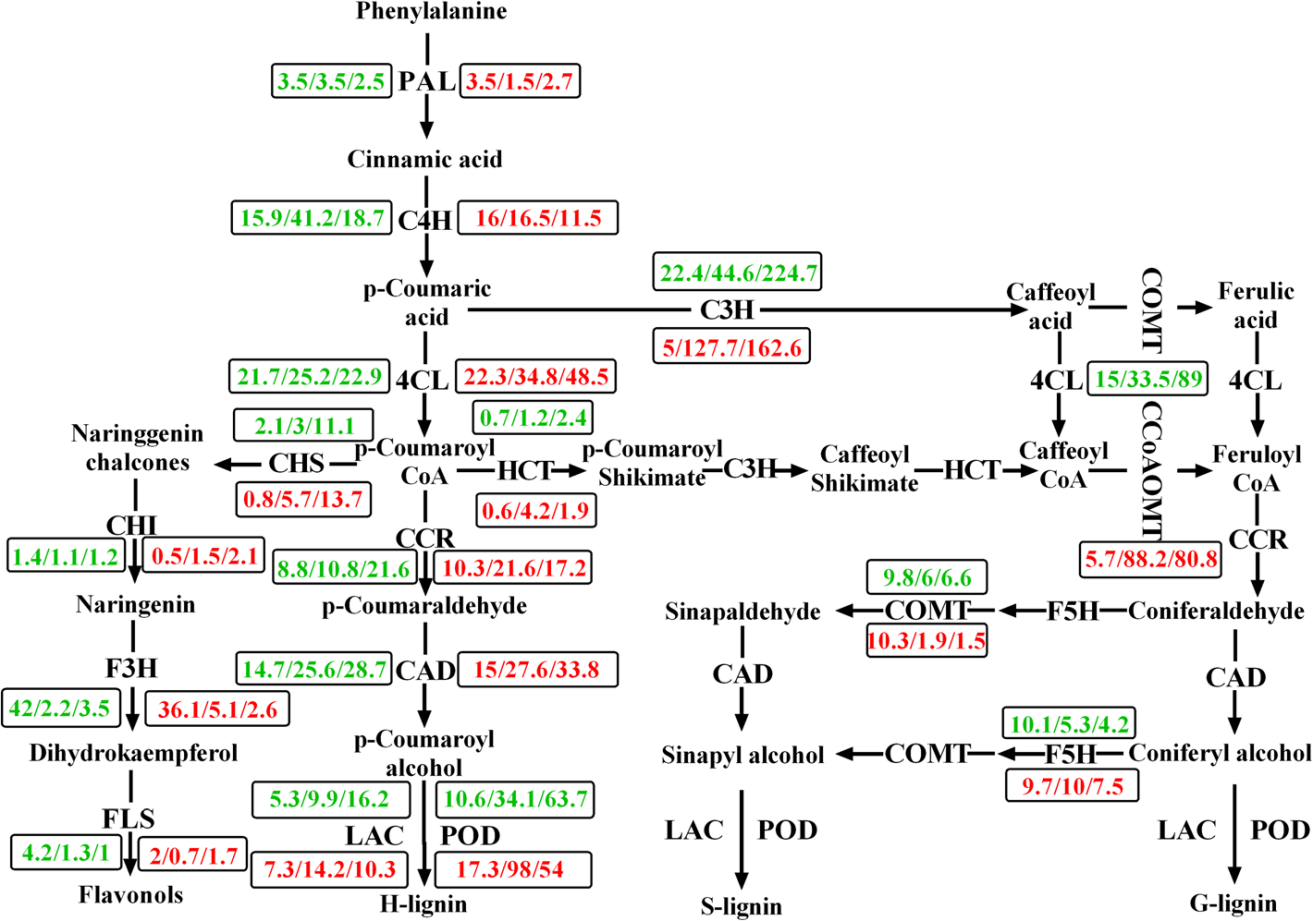
The expression of DEGs related with phenylpropanoid biosynthesis pathway. Red and blue numbers represent the DEGs from MBI8255 and CCRI36, respectively

During the process of plant growth and development, some phenylpropanoid metabolites differentially accumulate in particular cells or tissues, such as the lignins deposited in the xylem tissue and vascular bundles and the flavonoids concentrated in the vacuolar and wall compartments, which has been reported to play important roles in plant defense [49, 50]. The expression profiles of key enzyme genes involved in lignin and flavonoid biosynthesis were evaluated in this study (Fig. 9). The majority of up-regulated genes were related to lignin biosynthesis and the majority of down-regulated genes were related to flavonoid biosynthesis in the two lines during the preliminary stages of V991 infection (Additional file 3).

At the beginning of phenylpropanoid biosynthesis (from phenylalanine to p-coumaroyl CoA), the genes of phenylalanine ammonia-lyase (PAL), cinnamic acid.

4-hydroxylase (C4H), and 4-hydroxycinnamoyl-Coa ligase (4CL) exhibited almost no change from 0 DAI to 2 DAI in the two lines, except for down-regulation following the up-regulation of CCRI36 C4H and the continuous up-regulation of MBI8255 4CL. In the flavonoid biosynthesis pathway, the genes of chalcone synthase (CHS) and flavanone 3-hydroxylas (F3H) were persistently up-regulated, while the chalcone isomerase (CHI) and flavonol synthase (FLS) genes showed no significant change in the two lines, except for the continuous down-regulation of the FLS gene in CCRI36. With regards to lignin biosynthesis, continuously up-regulated expression patterns in the two lines were observed in the genes of cinnamoyl-CoA:NADPH oxidoreductase (CCR), coumarate-3-hydroxylase (C3H), caffeoyl-CoA O-methyl-transferase (CCoAOMT), hydroxycinnamoyl-CoA:shikimate hydroxycinnamoyltransferase (HCT), and cinnamyl alcohol dehydrogenase (CAD), while the caffeic acid O-methyltransferase (COMT) and coniferaldehyde/ferulate 5-hydroxylase (F5H) genes were down-regulated and exhibited little change from 0 DAI to 2 DAI. In MBI8255, hydroxycinnamoyl-CoA:shikimate hydroxycinnamoyltransferase (HCT), laccase (LAC), and POD genes were first up-regulated and then down-regulated. In contrast, the former exhibited no change in CCRI36, while the latter two were up-regulated.

### Expression profiling of the DEGs involved in oxidation-reduction processes

**Fig. 10:**
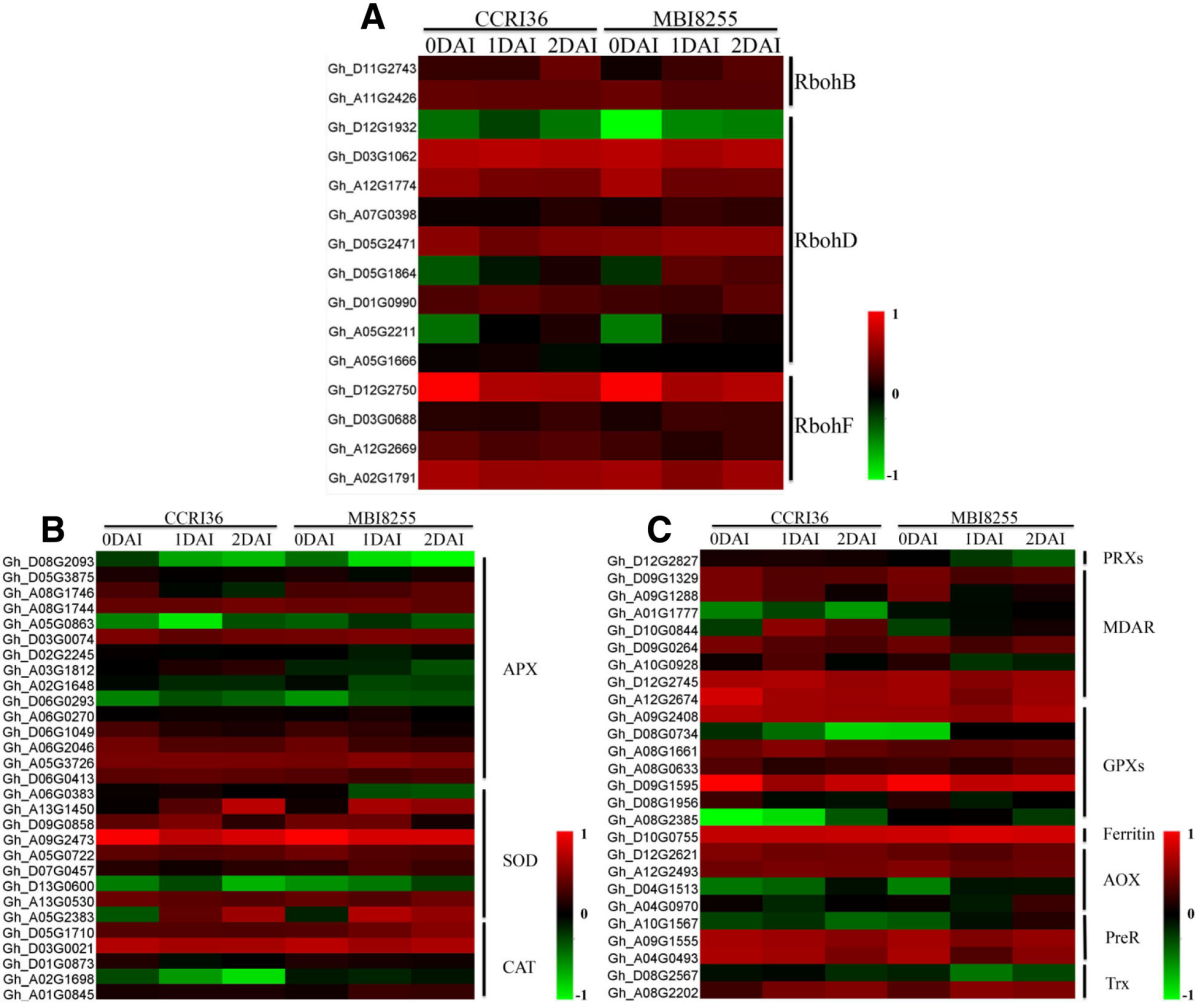
The heatmap analysis of genes related with oxidation-reduction process. **a** represents the expression patterns of genes related with ROS-producing pathways, (**b**) and (**c**) represent the expression patterns of genes related with ROS-scavenging pathways

Reactive oxygen species (ROS) have been reported to play significant roles in plant growth, development, and stress defense [51] and control redox homeostasis balance between ROS-producing and ROS-scavenging pathways. Based on the GO enrichment analysis in all of the DEGs, 1433 DEGs were found to participate in the oxidation-reduction process (GO: 0055114), which served as the basis for all the downstream analyses of ROS-related genes (Fig. 10).

NADPH oxidases, also known as respiratory burst oxidase homologs (RBOHs), play significant roles in the generation of ROS in plants [52]. In the present study, a total of 11 Rboh-like protein genes were identified, namely, 2 RbohB, 5 RbohD, and 4 RbohF in the oxidation-reduction process, with the former 2 mostly showing up-regulated patterns and the latter being down-regulated in response to V991 infection in the two lines.

Simultaneously, expression pattern analysis was also conducted on the DEGs involved in ROS scavenging pathways. Sixteen ascorbate peroxidase (APX) protein genes were identified that were mostly down-regulated in the two lines. There were eight SOD genes identified from our RNA-Seq data, most of which were first down-regulated and then up-regulated in CCRI36, but down-regulated in MBI8255. However, the two SOD genes annotated as copper/zine SOD 1 and manganese SOD 1 presented continuous up-regulation in CCRI36 and down-regulation following up-regulation in MBI8255, which was consistent with the aforementioned biochemical investigations of root SOD. Five catalase (CAT) genes were mostly down-regulated in CCRI36 and first down-regulated and then up-regulated in MBI8255, which corroborated the aforementioned CAT investigations. One glutaredoxin (GRX) gene exhibited no significant change in CCRI36, but presented a down-regulated pattern in MBI8255. In addition, the genes of eight monodehydroascorbate reductase (MDAR), seven glutathione peroxidase (GPX), and three peroxiredoxin (PrxR) genes were mainly down-regulated in CCRI36, but showed up-regulation following down-regulation in MBI8255. In both lines, one ferritin and three thioredoxin (Trx) genes presented up-regulated expression patterns, while most of the four alternative oxidase (AOX) genes were first down-regulated and then up-regulated. The correlation of all of the complicated and diverse changes in the above-mentioned genes with ROS-producing and ROS-scavenging pathways indicated that the oxidation-reduction process plays extraordinary roles in maintaining the redox homeostasis balance under V991 infection.

### Expression profiling of DEGs relevant to plant resistance

**Fig. 11:**
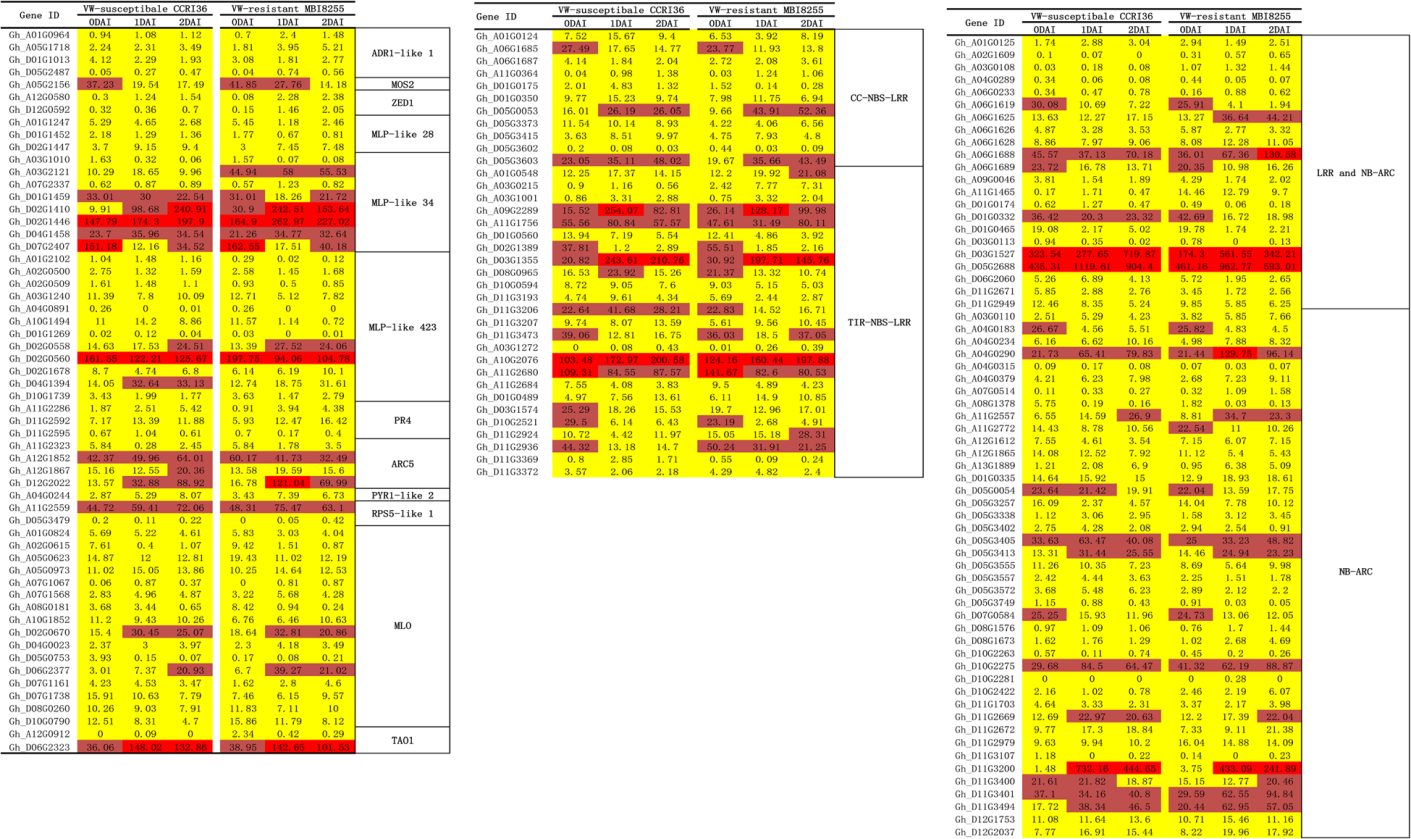
The expression pattern analysis of genes related with Defense response

Based on the GO enrichment analysis on all the DEGs, a total of 182 DEGs were enriched in the biological process of defense response (GO:0006952) and were further analyzed in combination with the functional annotation of *A. thaliana*, eventually obtaining 158 immunity-related DEGs (Fig. 11). In the present study, most DEGs associated with plant resistance to VW were annotated as R genes, which were classified into CC-NBS-LRR and TIR-NBS-LRR genes. Eleven CC-NBS-LRR protein genes were identified, of which Gh_D05G0053 and Gh_D05G3603 with relatively high levels gradually increased in the two lines (except for Gh_D05G0053 from 1 DAI to 2 DAI). There were 25 genes encoding TIR-NBS-LRR proteins, and four highly expressed genes were observed, namely, Gh_A10G2076, Gh_A09G2289, Gh_D03G1355, and Gh_A11G2680. The first and middle two genes were separately up-regulated and down-regulated after being first up-regulated in both lines, while the last gene showed continuous down-regulation in MBI8255 and up-regulation following down-regulation in CCRI36. In addition to the above-mentioned NBS-LRR proteins, we also found 22 genes encoding LRR and NB-ARC domain-containing disease resistance proteins and 42 genes encoding an NB-ARC domain-containing disease resistance protein in this GO term. Among the LRR and NB-ARC genes, Gh_D05G2688 and Gh_D03G1527 exhibited extremely high expression levels, with the former being first up-regulated and then down-regulated in the two lines, while the latter was up-regulated following down-regulation in CCRI36 and down-regulation following up-regulation in MBI8255. As for the NB-ARC genes, Gh_D11G3200 exhibited the highest expression and was first up-regulated and then down-regulated in the two lines.

Many other genes participate in plant resistance to VW, the expression patterns of which were further analyzed as follows. Activated disease resistance 1 (ADR1) genes*,* including ADR1*,* ADR1-like1, and ADR1-like2, were correlated with the regulation of the basal and ETI mediated defense in *Arabidopsis* [53]. Four ADR1-like1 genes were identified, namely, Gh_A01G0964, Gh_A05G1718, Gh_D05G2487, and Gh_D01G1013, in which the transcription patterns were mostly up-regulated in CCRI36 and down-regulated following up-regulation in MBI8255.

MOS2, also described as a D111/G-patch domain-containing protein, is essential for innate immunity in *A. thaliana* [54]. One MOS2 gene, Gh_A05G2156, was continuously down-regulated in the two lines, the expression level of which in MBI8255 was higher than in CCRI36, not only at 0 DAI but also at 1 DAI.

ZED1, also known as HOPZ-ACTIVARED RESISTANCE 1, is responsible for high temperature-dependent autoimmunity and growth retardation in zed1-D [55]. Two ZED1 genes, identified as Gh_A12G0580 and Gh_D12G0592, presented a gradually increasing expression level from 0 DAI to 2 DAI in the two lines.

Major latex protein (MLP)-like proteins belong to the Bet v 1 family, also described as the pathogenesis related 10 (RP 10)-like protein family, the expression patterns of which are related to defense or stress responses [56]. Gh_A01G1247, Gh_D01G1452, and Gh_D02G1447 were annotated as MLP-like protein 28 genes, of which the former two were up-regulated following down-regulation (except for down-regulation in Gh_A01G1247), whereas the latter was up-regulated from 0 DAI to 2 DAI in the two lines. Eight MLP-like 34 were identified, and the expression levels of Gh_D02G1410, Gh_D02G1446, and Gh_D07G2407 were relatively high in comparison to the remainder of the genes, with the former two being up-regulated and the latter being first down-regulated and then up-regulated in response to V991 infection in CCRI36 and MBI8255. There were 12 MLP-like 423 genes, and Gh_D02G0560 had extremely high RPKM values relative to the remainder, indicating up-regulation following down-regulation in both lines.

Three genes related to *Arabidopsis* pathogenesis-related 4 [57] were found, and the transcript level of Gh_D11G2592 was relatively high compared to Gh_A11G2286 and Gh_D11G2595, which were first up-regulated and subsequently down-regulated in CCRI36. In contrast, these were continuously up-regulated in MBI8255, obtaining higher expression levels in the VW-resistant line than the VW-susceptible line at 2 DAI.

A resistance (R) gene in rice belonging to P-loop containing the nucleoside triphosphate hydrolases superfamily protein has been implicated in the signaling pathways of the rice blast (*Magnaporthe grisea*) resistance reaction [58]. Four P-loops containing nucleoside triphosphate hydrolases superfamily protein genes, including Gh_A11G2323, Gh_A12G1867, Gh_A12G1852, and Gh_D12G2022, were identified, and the former two presented up-regulation after down-regulation, while the latter two were gradually up-regulated in CCRI36. In contrast, the first gene was up-regulated following down-regulation, the second and forth were down-regulated after being up-regulated, while the third was continuously down-regulated in MBI8255.

Pyrabactin resistance (PYR)/PYR1-like (PYL)/regulatory components of ABA receptors (RCAR) belong to the ABA receptor family, and ABA plays significant roles in plant abiotic stress resistance [59]. One PYL2 gene, Gh_A04G0244, was up-regulated in CCRI36, but was down-regulated after being up-regulated in MBI8255.

Resistance to Pseudomonas syringae 5 (RPS5), encoding the coiled-coil nucleotide-binding site leucine-rich repeat (CC-NBS-LRR) domain, recognizes the Avirulence effector protein Pseudomonas phaseolocola B (AvrPphB) from *Pseudomonas syringae* [60, 61]. Two RPS5-like genes were found in the results, namely, Gh_A11G2559 and Gh_D05G3479, of which the former with higher FPKM values than the latter was up-regulated in CCRI36, but down-regulated following up-regulation in MBI8255.

The mildew locus O (MLO) gene family, ubiquitously found in most land plants, encodes seven transmembrane (TM) domain proteins that participate in the regulation of defense and cell death in response to biotic and abiotic stress [62]. Fifteen genes annotated as MLO family proteins demonstrated diverse expressional abundances. Gh_D02G0670 and Gh_D06G2377 had relatively high RPKM values and were first up-regulated and then down-regulated in the two lines, except for the latter, which was consistently up-regulated in CCRI36. Interestingly, the expression levels in MBI8255 were higher than in CCRI36, except for Gh_D02G0670 at 2 DAI.

The target of AvrB operation 1 (TAO1), belonging to the TIR-NB-LRR protein family, was reported to contribute to disease resistance in response to the *Pseudomonas syringae* effector AvrB in *A. thaliana* [63]. Two TAO1 genes were identified, and Gh_D06G2323 was first up-regulated and then down-regulated in the two lines and exhibited significantly higher expression levels than Gh_A12G0912.

### Validation of the RNA-Seq results by quantitative real-time (qRT)-PCR

**Fig. 12:**
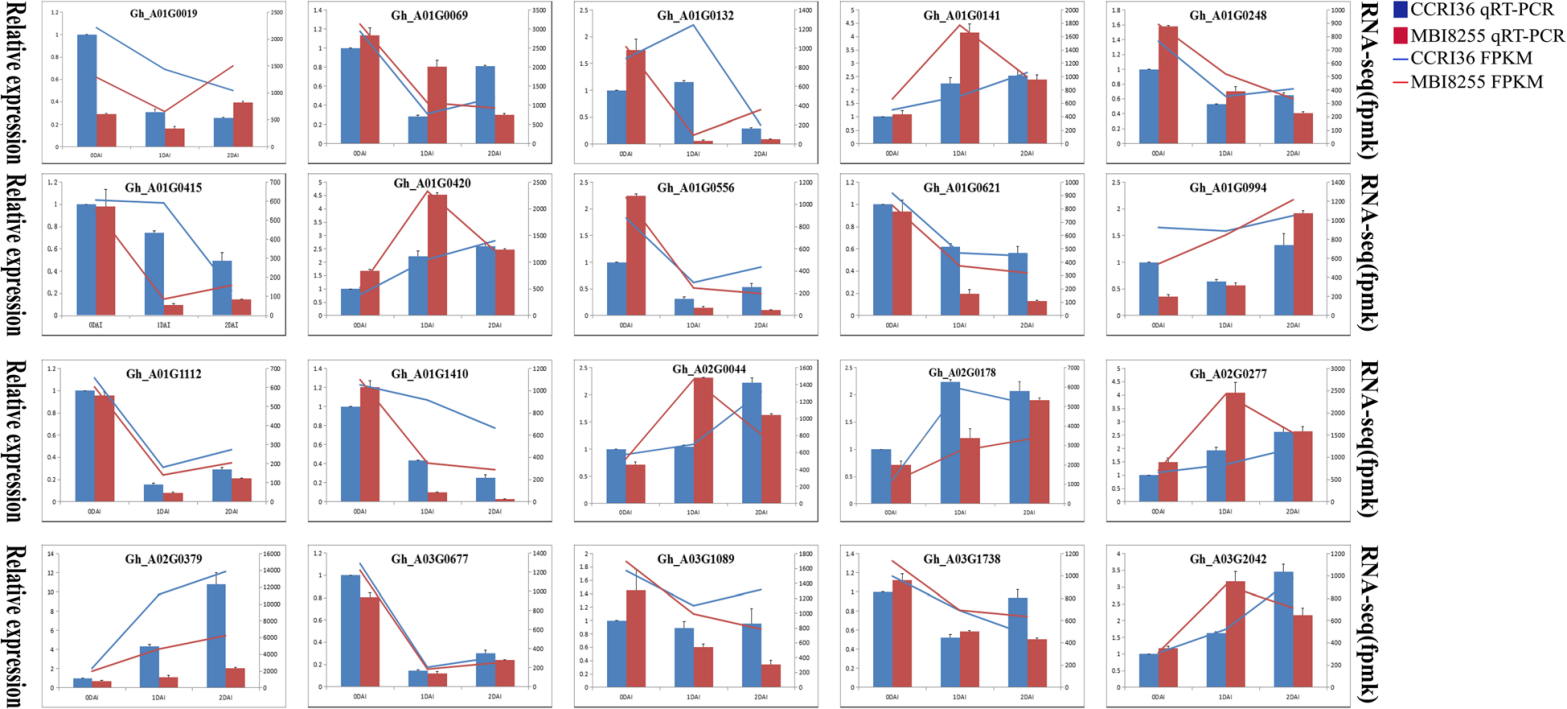
Validation of RNA-seq data by qRT-PCR. Columns indicate the results of qRT-PCR, and zigzag lines indicate the results of transcriptome sequencing.

To confirm the reliability of our RNA-Seq results, 20 DEGs were randomly selected for qRT-PCR (Fig. 12). The primer sets are listed in Table 4. The reference gene was the house-keeping β-Actin gene, and the expression patterns of the 20 genes in the qRT-PCR results were highly consistent with the transcriptome sequencing data, which further supported the reliability of our RNA-Seq data.

**Table 4:** Primers used for qRT-PCR validation

Gene ID	Forward 5`-3`	Reverse 5`-3`
Gh_A01G0019	CGTCATCGGCCACAAGAAAC	CATGTAAGCCACCGCCCTTA
Gh_A01G0069	TTGGGTGGATGAAACCCGAG	GACGGACTTCCTCCGATTC
Gh_A01G0132	GCCAATAACAAACTCGGCGG	CACCCGAAGGAACAATCCCA
Gh_A01G0141	GGTTTTCAAGGGTCTGGGCT	CGCTACTTTCTCCCTTGGCT
Gh_A01G0248	ATGCGGAGGAGGCTTGTAG	TCTTTCGTTCCGGCTTGGT
Gh_A01G0415	CAGGGCAAGAAAAGCAGTCC	GTTTCAGACCCACCACACCA
Gh_A01G0420	CCGATGGTTGGTTGCTGAAC	GAAGTGGGAGTCAAGAGCC
Gh_A01G0556	CTGGTGGAACTTCTTACACGC	CCTGCTGTCTGTGGTCTAC
Gh_A01G0621	CAGCATTTGAGTGGACAGC	ATGAACACCGACAAGCCGAA
Gh_A01G0994	CGCCACTCTACGATCCTTCC	CCAAACCTTGCGCCGTTAAA
Gh_A01G1112	CCCAAGTTAGCATGTTGAAGGC	GGGAAACGATCAAAGAGAAGCC
Gh_A01G1410	TGACTCTCTTCGGCGTCAAC	GCCATTTTGCCACCACTGAG
Gh_A02G0044	TGGTACGGAGACTGTGTGGTA	TCACGTAATCCCAGATCCTCG
Gh_A02G0178	CTCGTTGCACAAAGTGGTGG	TTTACGCCCTGGTTTCTCCC
Gh_A02G0277	GGGTTCTATGTGGTGGCTC	CCACAACCTTAGCACCCCAA
Gh_A02G0379	TTATCAGCCTCCGAAGAAGCC	TGGAGGGTGTCCTGGATAGT
Gh_A03G0677	AACCTCCTTCCATTCCACGC	TGCTTATGCCCCTTCCGATG
Gh_A03G1089	GGCGACTTGCAGAAAGAAAGG	CAGTTTTTCCCTCCGCTGG
Gh_A03G1738	CAACCGAGGAACGCAGGATA	CTCCCCTTCCGCTTGCTTTA
Gh_A03G2042	GCTTCCGGTCAAGTGGAGAA	CTCACGCGCTTCATCCCTAT
Gh_A01G0019	CGTCATCGGCCACAAGAAAC	CATGTAAGCCACCGCCCTTA

## Discussion

### Experimental conditions and phenotypic evaluation

In the present study, VW resistance evaluations were conducted on the CSSL, its recurrent parent, and the two controls under the artificial greenhouse, natural field, and disease nursery tests. The disease index can be calculated based on the number of leaves with symptoms of necrosis and chlorosis. Although three replications in the greenhouse tests were carried out with different numbers of paper cups (five seedlings per cup; the first replication included five cups, the second replication included seven cups, and the third replication contained 10 cups), the total number of seedlings for either the resistant control (Zhongzhimian2) or the susceptible control (Jimian11) was sufficient for the disease evaluation. This not only increases the accuracy, but also reduces the experimental error. Based on the greenhouse results, the DI values of MBI8255 and CCRI36 were relatively close to those of Zhongzhimian2 and Jimian11 at 15 DAI, respectively. A similar phenomenon was observed at 30 DAI, even though higher DI values were observed than at 15 DAI, which indicated that the selected CSSL and its recurrent parent were separately resistant and susceptible to V991.

In the natural field tests, the VW resistance evaluations were performed on the four lines as in the greenhouse investigations in two different locations (one replication in Anyang and one replication in Xinjiang), and were investigated at the flowering and maturation stages. The two resistant lines (MBI8255 and Zhongzhimian2) and two susceptible lines (CCRI36 and Jimian11) in Anyang presented relatively close DI values at both the flowering and mature stages. A similar trend was observed in Xinjiang, whereby MBI8255 exhibited VW resistance, while CCRI36 demonstrated VW susceptibility in the natural environment. The four lines in Anyang showed higher DI values than those in Xinjiang at the flowering stage, but presented relatively low DI values in Anyang at the mature stage, except in CCRI36. These variations in the field results could be attributed to a variety of factors, such as the infection of plants by a combination of VW strains, different locations with diverse quantities and virulence levels of soil fungus, and various developmental and environmental conditions [64]. For the disease nursery tests, only one replicate of MBI8255, CCRI36, and Jimian11 was conducted in Kaifeng, while the susceptible control showed 56.77% DI, which was slightly higher than in either the greenhouse or field tests. Although presenting a more similar DI to that at 15 DAI or the flowering stage of the greenhouse and field tests, MBI8255 and CCRI36 separately exhibited resistance and susceptibility to VW, which might be due to the insufficient number of sampled plants. In combination with the greenhouse, field, and disease nursery results, the selected CSSL, MBI8255, proved to be resistant to VW despite showing a slightly higher DI than the resistant control, Zhongzhimian2. Conversely, the recurrent parent, CCRI36, was susceptible to VW despite demonstrating a slightly lower DI than the susceptible control, Jimian11, which provided a solid foundation for further biochemical tests and transcriptome sequencing.

### Biochemical mechanisms of resistance to V991 infection

As a result of long-term interactions with diverse pathogens, plants have evolved many strategies to prevent damage or invasion by microbes, including plant physical defense responses and physiological and biochemical resistance, of which the regulation of antioxidant enzymes and the synthesis of biochemical substances is of great significance. ROS have been shown to mediate signal transduction and regulate homeostasis as key secondary messengers, high concentrations of which could result in membrane damage and the destruction of cellular organelles and biomolecules, ultimately causing cell death [65]. In order to repair the damage caused by ROS in response to pathogen infection, plants enhance the activities of antioxidative enzymes, such as CAT, SOD, POD, and PPO. In addition to the above-mentioned enzymes, PAL is also an essential enzyme that is correlated with the synthesis of lignin and suberin and plays a key role in the regulation of the synthesis of salicylic acid (SA) and the establishment of systemic acquired resistance (SAR) [66]. With regards to the VW caused by *V. dahliae*, the POD, PAL, and PPO activities were positively correlated with VW resistance in eggplant [67], and the former two were strengthened after inoculation with the V991 strain in cotton [43]. To evaluate the relationships between key enzymes and VW resistance, three protective enzymes (CAT, SOD, and POD) and two defense enzymes (PAL and PPO) were selected and tested on root samples collected at 0, 1, and 2 DAI in MBI8255 and CCRI36. According to the activity results of the protective enzymes (Fig. 2), the activities of the VW-resistant and VW-susceptible lines at the same stage showed no significant difference either in root CAT or root SOD, which indicated that no correlation between VW resistance and CAT activity or SOD activity was detected in the present study. However, significant differences were observed in POD activity between MBI8255 and CRI36 at the same stage, except at 1 DAI, which implied a close relationship between POD activity and VW resistance. Furthermore, POD activity in MBI8255 was significantly higher than in CCRI36 at 0 and 2 DAI, which indicated that in comparison to the susceptible recurrent parent, the resistant CSSL possessed higher POD activity regardless of V991 infection. With regards to the two defense enzymes, only PPO activity in the two lines at 2 DAI differed significantly, while the VW-resistant line presented higher PAL and POD activities than the VW-susceptible line, except for PAL activity at 1 DAI. This indicated a clear correlation between VW resistance and POD activity, and furthermore, that the resistant line had higher PAL and POD activity in response to V991 infection. Based on the above results, the POD and PPO activities were positively correlated with VW resistance, which was consistent with the findings of a previous study [43, 67]. However, sufficient evidence is lacking to prove a close correlation between PAL activity and VW resistance, although higher activities were identified in the resistant line, which did not corroborate previous studies. One reason might be related to the complicated background of the selected CSSL, which should be addressed in further research.

Some biochemical substances in plants provide energy for the self-defense response or are the products of damage and injury caused by pathogen invasion, including MDA [68, 69], proline [70, 71], and soluble sugar [72], which are correlated with plant resistance. As the final product of lipid peroxidation, the root MDA contents in the two lines showed significant differences at the same stage and a down-regulated pattern, except at 0 DAI, and presented higher contents in MBI8255, which might indicate a negative correlation with VW resistance. However, an obvious correlation between VW resistance and the contents of proline, soluble sugar, and soluble protein was not detected, and no significant differences were found between either the two lines at the same stage, or between MBI8255 or CCRI36 at the different stages. Our results corroborated previous studies [43, 67], thereby providing useful information for the further analysis of the combination with our RNA-Seq data.

### Gene expression variation in response to V991 infection

RNA-Seq, as a powerful tool focused on global gene expression at the transcriptome level, can reveal the mechanisms of particular biological processes and was utilized to identify the key candidate genes or significant signaling pathways in response to VW in the present study. Eighteen root samples derived from the VW-resistant MBI8255 and VW-susceptible CCRI36 were collected at 0, 1, and 2 DAI of V991 infection. Library construction and transcriptome sequencing of these samples produced a total of 908.547 million clean reads (approximately 136.27 Gb data) with more than 91.38% of the Q30 value and over 43.44% of the GC content. No less than 87.69% of the clean reads were successfully mapped to the reference genome and were subsequently subjected to assembly, ultimately obtaining 77,412 genes, including 6934 novel genes. PCC analyses were used to confirm the relationships of the different samples, and high similarities were found among the three replicates of each sample and also in the samples from the same stages, which confirmed the reliability of our RNA-Seq data.

Through multiple comparisons between the samples, 23,180 genes (1816 novel genes) were differentially expressed in response to V991 infection, with more down-regulated genes than up-regulated genes observed. Adopting STEM analysis to investigate the temporal expression patterns of the total DEGs, three profiles (*P* ≤ 0.05) per line were identified, and GO and KEGG enrichment analysis were performed on up-regulated profile 7 and down-regulated profile 2, which exhibited the same patterns. In the two profiles, metabolic process and cellular process, binding and catalytic activity, and cell part and cell were the most abundant subcategories in biological process, molecular function, and cellular component, respectively. The genes clustered into the same GO terms in MBI8255 and CCRI36 showed opposite patterns, and there were more enriched genes of CCRI36 over those of MBI8255 in profile 7 while presenting less enriched genes of CCRI36 than those of MBI8255 in profile 2. Based on the KEGG results, only two pathways, namely, phenylpropanoid biosynthesis and plant hormone signal transduction, in the two profiles or in the two lines, were significantly enriched, and more genes were clustered in CCRI36 than in MBI8255 in the up-regulated expression pattern (profile 7), while more genes were down-regulated in MBI8255 than in CCRI36 (profile 2). The consistency between the GO and KEGG results might be due to the diverse strategies adopted in response to V991 infection by the different resistant or susceptible lines. With regards to the enriched metabolic processes in our results, secondary metabolism pathways were found to play particularly significant roles in response to V991 infection and they have been reported to participate in the abiotic stress response [73]. Furthermore, phenylpropanoid and flavonoid biosynthesis pathways have also been suggested to accumulate in response to pathogen infection, as indicated in a previous study [74].

### The phenylpropanoid metabolic pathway in response to V991 infection

The phenylpropanoid pathway in plants has been reported to participate in some abiotic stress responses, such as drought, salinity, and pathogen infection, and it serves as the main metabolic pathway for lignin biosynthesis [75]. As a major cell wall component, lignin acts as a main plant mechanical support structure and defense system in response to pathogen infection [76], the synthesis of which is subjected to the oxidative coupling of *p*-hydroxyphenyl (H), guaiacyl (G), and syringyl (S) monolignols. In this study, the majority of the key enzymes in the phenylpropanoid pathway were analyzed based on the combined RNA-Seq and qRT-PCR results. Diverse expression patterns were observed in response to V991 infection, mostly presented by the up-regulation of lignin biosynthesis-related enzymes rather than flavonoid synthesis-related enzymes, which might result from the antagonistic relationships between the two synthesis pathways. Nevertheless, our findings still indicate the positive effect of V991 infection on the phenylpropanoid pathway in CCRI36 and MBI8255, further promoting lignin biosynthesis.

During the synthesis pathways of G and S type lignins, most genes showed up-regulated expression patterns, including C3H, CCR, and CAD, while only one down-regulated gene, COMT, was identified, which represented the difficult shift from caffeic acid to ferulic acid during the synthesis of S and G type lignins. Furthermore, the up-regulated CCoAOMT, which transforms caffeoyl CoA to feruloyl CoA, might be efficiently utilized to obtain adequate lignin, as has been reported in switchgrass and duckweed [77, 78]. In general, the lignin biosynthesis pathway stimulated by V991 infection would produce a higher lignin content, which could combine with some other antioxidants to participate in the regulation of ROS production in the apoplast [76]. These results revealed the significant role of the phenylpropanoid metabolic pathway in response to V991 infection.

### Oxidation-reduction processes in response to V991 infection

In response to abiotic stress, ROS generation in plants can be activated by ROS-producing genes, resulting in oxidative stress and accordingly giving rise to ROS-scavenging pathways in order to maintain the ROS homeostatic balance. In the present study, a total of 1433 DEGs were found to participate in oxidation-reduction processes, derived from the GO analysis results of all the DEGs. RBOHs, identified as RbohB, RbohD, and RbohF in our study, have been reported to participate in the process of ROS production [52] and mostly showed up-regulated patterns from 0 DAI to 2 DAI, which indicated increased oxidative stress in response to V991 infection.

In order to eliminate the generated ROS, plants have evolved ROS scavenging pathways in response to damage caused to the cellular components by high oxidative stress. Among the antioxidant enzymes, SOD, as the first line of antioxidant defense, is responsible for transforming O_2_^−^ into H_2_O_2_, and subsequently APX and CAT further transform H_2_O_2_ into H_2_O with their corresponding compounds [79]. Under V991 infection, only two SOD genes showed up-regulated expression patterns in CCRI36, but presented down-regulation after up-regulation in MBI8255, which was consistent with the SOD results of the biochemical tests, indicating the significant roles of the two enzymes in ROS scavenging pathways. APX or CAT were mainly down-regulated in CCRI36, but were up-regulated following the down-regulation in MBI8255. Similar results were observed with regards to the activity changes of MDAR, GPXs, and PreR. In the two lines, in addition to the up-regulation and then down-regulation of AOX enzymes, ferritin and Trx were continuously up-regulated, which suggested their positive participation in ROS scavenging pathways. Therefore, under *V. dahliae* infection, ROS generation increased in both CCRI36 and MBI8255, subsequently leading to the activation of ROS scavenging pathways to protect the plants. In contrast, the resistant line exhibited relatively higher ROS scavenging-related enzyme activity than the susceptible line, which might be due to the resistant line possessing a relatively well-developed ROS scavenging system.

### Plant resistance in response to V991 infection

The process of pathogen infection starts from an influx of calcium and an oxidative burst triggered by PAMPs, and subsequently activates not only the MAPK pathway and calcium-dependent protein kinase, but also stomatal closure and transcriptional reprogramming, ultimately resulting in SA accumulation and callose deposition [79–81]. In order to respond to the invasions from various pathogens, plants have evolved two main defense mechanisms. Along with the recognition of PAMPs occurring on the extracellular surface of the host cell, PPRs together with their stimulation activate a basal immune response, namely, PTI [27, 28]. Upon successful pathogen invasion, the generated effectors are first delivered into the host cells, further suppressing the PTI, which can be recognized by specific R genes. ETI, the second-type immune reaction, is activated by the production of R genes. The induction of plant resistance to pathogens and HR are ultimately triggered following the special recognition of both PRRs and R genes [30, 31].

In the present study, the interactions of two lines, MBI8255, resistant to VW, and CCRI36, susceptible to VW, with *V. dahliae* were investigated with the aim of revealing the molecular mechanisms of the plant immune response to plant pathogens. Based on the results of GO enrichment analysis of all of the DEGs, a total of 158 immunity-related genes with *A. thaliana* annotations were clustered into the defense response GO term (GO: 0006952), and significantly different expression patterns were observed between the resistant and susceptible lines. Among the resistance genes, R genes were mostly identified from the results and could be classified into TIR-NBS-LRR and CC-NBS-LRR genes based on the different N-terminal domains [35, 36]. As numerous genes were annotated as R genes, only those with high-level expression were further investigated, although more genes encoding the second-type R domain were observed. With regards to the first-type R genes, up-regulated expression patterns were identified in the two highly expressed genes, Gh_D05G0053 and Gh_D05G3603, with the former showing higher expression in MBI8255 than in CCRI36, except at 0 DAI, while the latter presented no significant difference between the expression levels of the two lines at the same stage. There were more than twice as many second-type R genes than first-type R genes, and five high-expression genes were found, namely, Gh_D05G3603, Gh_A10G2076, Gh_A09G2289, Gh_D03G1355, and Gh_A11G2680. The first four of these genes showed up-regulation from 0 DAI to 1 DAI, while the latter one was continuously down-regulated during the preliminary stages of V991 infection, and, interestingly, was highly expressed at 0 DAI in the resistant line. In addition, there were 22 genes encoding LRR and NB-ARC domain-containing disease resistance proteins in this term, and the two high-level expressed genes, Gh_D03G1527 and Gh_D05G2688, showed the opposite expression patterns, with the former being first down-regulated and then up-regulated, while the latter was down-regulated following up-regulation in the two lines. Over 40 genes encoding NB-ARC domain-containing disease resistance proteins were identified, while only the relatively highly expressed Gh_D11G3200 was first up-regulated and then down-regulated in the two lines.

In addition to the above-mentioned R-related genes, there were many other resistance genes involved in PTI and ETI. Two genes annotated as ADR1-like 1, namely, Gh_A05G1718 and Gh_D01G1013, were relatively highly expressed, with the former showing continuous up-regulation in the two lines, while the latter was down-regulated in CCRI36 and up-regulated following down-regulation in MBI8255. In the two lines, one MOS2 gene (Gh_A05G2156) was persistently down-regulated, while two ZED1 genes, Gh_A12G0580 and Gh_D12G0592, were up-regulated. Four MLP-like genes with high-level expression, Gh_D02G1410, Gh_D02G1446, Gh_D07G2407, and Gh_D02G0560, were investigated, with the former two being up-regulated in CCRI36 and first up-regulated and then down-regulated in MBI8255, while the latter two showed up-regulation following down-regulation in the two lines. Both pathogenesis-related 4 (Gh_D11G2592) and PYR1-like 2 (Gh_A04G0244) were up-regulated in the two lines, and the same pattern was observed in the three genes encoding P-loop containing nucleoside triphosphate hydrolases superfamily proteins (Gh_A12G1852, Gh_A12G1867, and Gh_D12G2022) in CCRI36, with opposite expressions observed between the former and the latter two. The RPS5-like 1 gene, Gh_A11G2559, was up-regulated in CCRI36, but was first up-regulated and then down-regulated in MBI8255, as observed in one highly expressed MLO gene, Gh_D06G2377. The same expression patterns were identified between other high-level MLO genes (Gh_D02G0670) and TAO1 (Gh_D06G2323), which showed up-regulation following down-regulation in the two lines. Interestingly, higher-level expression was observed in the resistant lines over those in the susceptible line at 0 and 1 DAI, although diverse expression patterns were identified in the above immunity-related genes, which suggested that the greater resistance to VW might result from the better defense mechanisms in cotton.

## Conclusion

In this study, artificial greenhouse, natural field, and disease nursery tests were conducted to confirm the selected CSSL (MBI8255) and its recurrent parent (CCRI36) together with two controls (Zhongzhimian2 and Jimian11), resulting in VW-resistant MBI8255 and VW-susceptible CCRI36. Subsequently, the two lines were separately subjected to biochemical tests and RNA-Seq, using root samples collected during the preliminary stages of V991 infection (0, 1, and 2 DAI). In terms of the biochemical tests, the activities of POD and PPO were positively correlated with VW resistance, while a negative correlation was detected between MDA content and VW resistance. Additionally, a total of 77,412 genes, including 6934 novel genes, were identified in our RNA-Seq data, further leading to the identification of 23,180 DEGs through multiple comparisons. Following gene temporal expression pattern analysis, two identical profiles (*P*-value ≤0.05) derived from the DEGs of the two lines were further investigated by GO and KEGG enrichment analysis, which mostly clustered into metabolic process and cellular process in biological process, binding and catalytic activity in molecular function, and cell part and cell in cellular component, while significantly participating in the pathways of phenylpropanoid biosynthesis and plant hormone signal transduction. Finally, expression profiling of the DEGs related to the phenylpropanoid metabolic pathway, oxidation-reduction process, and plant resistance was carried out to identify the key candidate genes or pathways, which provided an abundance of information for revealing the mechanisms of VW resistance. The results of this study facilitate further research into cotton VW resistance by not only providing resistant material for cotton breeding, but also by improving our understanding of the mechanisms of VW resistance.

## Methods

### Preparation of *V. dahliae* and plant material

The *V. dahliae* fungus isolate used in this study was the highly aggressive defoliating V991. V991 was first cultured on solid potato dextrose agar (PDA) medium for 7 d at 25 °C and then transferred to Czapek liquid medium to obtain conidia, after which it was placed in an incubated shaker (Haerbin Donglian Electonics, China) with a shaking speed of 150 rpm/min in darkness for 7–14 d at 25 °C. The concentration of the spore suspension was diluted to about 1 × 10^7^ spores/mL with sterile water for the root inoculation, which was assessed using a hemocytometer [6, 16].

The CSSLs constructed by our lab were derived from the hybridization between CCRI36 and Hai 1 in 2003 at the Institute of Cotton Research of the Chinese Academy of Agriculture Sciences (CRICAAS; Anyang, Henan province, China), in which the population was successfully developed through multiple generations of backcrossing and selfing, as described elsewhere [82]. CCRI36 developed by CRICAAS is a *G. hirsutum* cultivar with wide planting areas and high-yield fiber products, while Hai 1 stored by CRICAAS is a *G. barbadense* cultivar with excellent fiber quality and high resistance to Verticillium wilt [24]. Based on VW resistance investigations in multiple locations for years, MBI8255 and CCRI36 were found to be stably resistant and susceptible to VW, respectively. Two controls, Zhongzhimian2 and Jimain11, were selected as VW-resistant and VW-susceptible controls during the subsequent greenhouse and field tests.

### Greenhouse tests

MBI8255 and CCRI36 together with the two controls (Zhongzhimian2 and Jimian11) were selected for the VW resistance tests in an artificial greenhouse located at the Institute of Cotton Research, Chinese Academy of Agricultural Sciences (ICR-CAAS). After disinfecting with 75% ethanol for 30 s and 2.5% sodium hypochlorite for 10 min, the seeds were washed with sterile water 3–5 times and then subsequently placed into paper cups filled with sterilized sand and vermiculite in a proportion of 4:6. There were 7–10 seeds that were planted in each cup for germination, and 5 seedlings per cup that exhibited good and synchronous growth were reserved for further *V. dahliae* infection. Three replications were performed: (1) five paper cups from March 28, 2015 to May 30, 2015; (2) seven paper cups from August 26, 2015 to October 28, 2015; and (3) 10 paper cups from August 28, 2015 to October 30, 2015. When the first true leaf was open and flat, each paper cup was placed in a paper tray, the bottom of which had been removed with scissors, together with 10 mL of 1 × 10^7^ spores/mL conidial suspension.

Phenotypic identification of VW resistance was separately carried out at 15 and 30 DAI, and each seedling was evaluated according to a severity rating system (from 0 to 4) based on the disease symptoms of the plant leaves [83]. Specifically, 0 indicates healthy plant without disease symptom; 1 indicates less than 25% leaves with disease symptoms; 2 indicates 25–50% leaves with disease symptoms; 3 indicates 50–75% leaves with disease symptoms; and 4 indicates over 75% leaves with disease symptoms and even completely defoliated or dead plants. The DI was adopted to calculate the VW resistance using the following formula:

$$ \mathrm{DI}=\left[\sum \left(\mathrm{N}\mathrm{i}\times \mathrm{i}\right)/\left(\mathrm{N}\times 4\right)\right]\times 100 $$

where i is the disease grade from 0 to 4, Ni is the number of plants with the corresponding disease grade, and N is the number of total plants investigated for each material.

### Natural field and disease nursery tests

The natural field investigations of MBI8255, CCRI36, and the two controls were conducted on two different experimental farms: one replication in Anyang (Henan Province) and one replication in Shihezi (Xinjiang Province). Under natural conditions, the materials planted in the field for VW resistance investigations were infected with a mixture of *V. dahliae* isolates*.* The phenotypic identification of VW resistance was performed at the flowering and maturity stage in 2015, and DI was calculated using the above-mentioned formula.

With the help of Professor Cai Yingfan, the disease nursery tests of MBI8255, CCRI36, and Jimian11 were conducted by the State Key Laboratory of Cotton Biology, Henan Key Laboratory of Plant Stress Biology, School of Life Science, Henan University. The phenotypic data were collected at the maturity stage in 2015, and DI was calculated using the formula described above.

### Sample collection

Based on **t**he VW-resistance results of the artificial greenhouse, natural field, and disease nursery tests, MBI8255 and CCRI36 were separately selected as the VW-resistant and VW-susceptible lines for further RNA-Seq and biochemical tests. The sterilized seeds were planted in three plastic trays filled with sterilized sand and vermiculite (4:6) and placed in a constant temperature incubator, with three biological replications. The culture conditions were set as follows: 25 °C, 60–70% relative humidity, and a photoperiod of 14 h/10 h (day/night). When the first true leaf was open and flat, the seedlings were removed from the plastic trays and inoculated via root-dipping into either the 1 × 10^7^ spores/mL conidia suspension or sterile water (for mock inoculation). After inoculation for 60 s, the seedlings were transferred back to the plastic trays in the constant temperature incubator. The root samples were harvested at 0, 1, and 2 DAI, quickly frozen in liquid nitrogen, stored at − 80 °C, and divided into two portions: one for RNA-Seq with three replications and another for the biochemical tests with three replications.

### Biochemical tests

Protective enzymes relevant to VW resistance were selected to investigate the activity changes during the preliminary stages of V991 infection (0, 1, and 2 DAI), including CAT, SOD, and POD. Fresh roots (0.1 g) per sample were ground in liquid nitrogen and suspended in 0.9 mL phosphate buffer (pH 7.4) or saline solution. The 10% tissue homogenate of the CAT samples was centrifuged at 2500 rpm for 10 min, while those of the POD and SOD samples were centrifuged at 3500 rpm for 15 min. The obtained supernatants were collected to determine the activities of CAT, POD, and SOD using commercially available kits from the Nanjing Jiancheng Bioengineering Institute (Nanjing, China), and were separately measured using a spectrophotometer at 405, 420, and 550 nm.

The defensive enzymes relevant to VW resistance, namely, PAL and PPO, were subjected to activity tests at 0, 1, and 2 DAI. Fresh roots (0.1 g) per sample were ground in liquid nitrogen and subsequently suspended in 0.9 mL extracting solution on ice. The tissue homogenate of the PAL sample was centrifuged at 4 °C and 10,000 rpm for 10 min, while the PPO sample was centrifuged at 4 °C at 8000 rpm for 10 min. The obtained supernatants were further processed according to the instructions of the PAL and POD assay kits from the Nanjing Jiancheng Bioengineering Institute and were measured spectrophotometrically at 209 and 525 nm.

Biochemical substances relevant to VW resistance, such as MDA, soluble sugar, soluble protein, and proline, were selected to assess the content changes during the V991 infection process. Fresh roots (0.1 g) were ground in liquid nitrogen and then suspended in 0.9 mL solution of phosphate buffer (pH 7.4) or saline for the MDA, soluble protein, and proline determinations. The homogenate of the MDA samples was centrifuged at 2500 rpm for 10 min, while that of the soluble protein and proline samples were centrifuged at 3500 rpm for 15 min. With regards to the content of soluble sugar, 0.1–0.2 g fresh roots were ground with 1 mL distilled water and then cultured in a boiling water bath for 10 min. After cooling, the tissue homogenate of the soluble sugar was centrifuged at room temperature at 8000 rpm for 10 min. The collected supernatants were further used for the content tests according to the instructions of the MDA, soluble sugar, soluble protein, and proline assay kits from the Nanjing Jiancheng Bioengineering Institute, and were separately measured spectrophotometrically at 532, 620, 595, and 520 nm. Three biological replications per sample were performed.

### RNA extraction, library construction, and transcriptome sequencing

According to the instruction manual of the RNAprep Pure Plant Kit (Tiangen, Beijing, China), the total RNA per sample was extracted from the frozen roots. The extracted RNA was first subjected to degradation and contamination detection by 1% agarose gel electrophoresis and integrity assessment by the RNA Nano 6000 Assay Kit of the Bioanalyzer 2100 system (Agilent Technologies, CA, USA), subsequently being calibrated by the Qubit® RNA Assay Kit in a Qubit® 2.0 Fluorometer (Life Technologies, CA, USA). High-quality RNA (3 μg) per sample was required for cDNA library construction following the manufacturer’s recommendations of NEBNext® Ultra™ RNA Library rep Kit for Illumina® (NEB, USA), and index codes were added to attribute the sequences to each sample. After being purified from total RNA by poly-T oligo-attached magnetic beads, mRNA was fragmented into 150–200 nt RNA inserts, which were used to synthesize the first-strand cDNA by random hexamer primer and M-MuLV Reverse Transcriptase (RNase H^−^) and the second-strand cDNA by DNA Polymerase I and RNase H. End-repair and adaptor ligation were performed on the double-stranded cDNA. The suitable fragments were purified with an AMPure XP system (Beckman Coulter, Beverly, USA) and enriched by PCR amplification. Subsequently, PCR products were purified using the AMPure XP system and assessment of library quality was performed on the Agilent Bioanalyzer 2100 system. For cluster generation, a cBot Cluster Generation System was used for clustering of the index-coded samples by the TruSeq Cluster Kit v3-cBot-HS (Illumina). In total, 18 cDNA libraries constructed from 18 RNA-Seq samples (two cotton lines at 0, 1, and 2 DAI with three replications) and were sequenced on a flow cell with an Illumina HiSeq™ 2500 sequencing platform.

### Data quality control and read mapping to the reference genome

The raw data in FASTQ format were first processed through in-house Perl scripts, resulting in read sequences and their corresponding base qualities. The clean data were obtained by filtering adapters and low-quality reads with poly-*N* > 10% or Q20 < 20% according to the Illumina pipeline, and the Q30 and GC content were subsequently calculated.

The high-quality clean reads were mapped to *G. hirsutum* as the reference genome [40] together with gene model annotation files*,* which were downloaded from the CottonGen database (http://www.cottongen.org). The index of the *G. hirsutum* genome was built by Bowtie v2.2.3 and the paired-end clean reads were aligned to the reference genome (*G. hirsutum*) using TopHat v2.0.12 [84].

### DEG analysis

In order to quantify the gene expression levels, the read numbers mapped to each gene were counted by HTSeq v0.6.1, and then the FPKM value was employed to calculate the expression level of each gene based on the length of the gene and the read counts mapped to this gene. As the most commonly utilized method for estimating gene expression levels at present, FPKM considers the effect of sequencing depth and gene length for the read counts at the same time [70].

The DESeq R package (v1.18.0) was used to identify differential gene expression between the control and treatment, which provided statistical routines for identifying the differential expression in the digital gene expression data using a model based on the negative binomial distribution. To manage the FDR, Benjamini and Hochberg’s approach was performed to adjust the obtained *P*-values. DEGs were set as those genes with an adjusted *P*-value ≤0.01 as determined by DESeq.

GO enrichment analysis of the DEGs was performed by the GOseq R package with a corrected *P*-value ≤0.5 as the cutoff. KOBAS software was used to test the statistical enrichment of the DEGs in the KEGG pathways.

### Comparison of the expression patterns of the DEGs

STEM software (Carnegie Mellon University, USA) was used to identify the temporal expression patterns of the DEGs in response to V991 infection (0, 1, and 2 DAI). GO and KEGG enrichment analysis was also employed to identify the potential functional genes or to characterize the expression profiles identified by STEM analysis.

### Validation of RNA-Seq by qRT-PCR

In order to verify the reliability of the transcriptome data, qRT-PCR was performed on 20 randomly selected DEGs with three biological and technical replicates for each sample. The Primer-BLAST online tool of NCBI was used to design the specific primers of the selected DEGs. The cDNAs were synthesized using a TranScript All-in-One First-Strand cDNA Synthesis SuperMix for qPCR Kit (Transgen Biotech, Beijing, China). qRT-PCR was performed following the protocol of TransStart Top Green qPCR SuperMix kit (Transgen Biotech, Beijing, China) on the ABI 7500 fast Real-Time PCR System (Applied Biosystems, USA). The β-Actin housekeeping gene was used as the reference to normalize the relative expression levels, with the following primer sequences: F: 5’-ATCCTCCGTCTTGACCTTG-3′ and R: 5’-TGTCCGTCAGGCAACTCAT-3′. The qRT-PCR carried out in a 20 μL system at the following conditions: one cycle of 94 °C for 30s; 40 cycles of 94 °C for 5 s, 60 °C for 34 s, and one cycle of 60 °C for 60s. The relative gene expression level was quantified using the 2^-ΔΔCt^ method [85].

## Additional files

Additional file 1: Differentially expressed genes between the samples. (XLS 3625 kb)

Additional file 2: Temporal expression of genes in two materials. (XLS 1123 kb)

Additional file 3: Gene names and their expression levels related with phenylopropanoid metabolic pathway. (XLS 20 kb)

## Abbreviations

ADR1: Activated disease resistance 1
AOX: Alternative oxidase
APX: Ascorbate peroxidase
CAT: Cayalse
CSSLs: Chromosome Segment Substitution Lines
DAI: Days after inoculation
DEGs: Differentially expressed genes
DI: Disease index
ETI: Effector-triggered immunity
FDR: False discovery rate
FPKM: Fragments per kb per million of the mapped reads
GPX: Glutathione peroxidase
GRX: Glutaredoxins
HR: Hypersensitive response
MAS: Marker assisted-selection
MDA: Malondialdehyde
MDAR: Monodehydroascorbate reductase
MLO: Mildew locus O
MLP: Major latex protein
PAL: Phenylalanine ammonia-lyase
PAMPs: Pathogen-associated molecular patterns
PCC: Pearson Correlation Coefficient
PCD: Programmed cell death
PDA: Potato dextrose agar
POD: Peroxidase
PPO: Polyphenol oxidase
PRRs: Pattern recognition receptors
PrxR: Peroxiredoxin
PTI: PAMP-triggered immunity
qRT-PCR: Quantification real-time PCR
QTL: Quantitative trait locus
RBOHs: Respiratory burst oxidase homologs
ROS: Reactive oxygen species
RPS5: Resistance to pseudomonas syringae 5
SOD: Superoxide dismutase
STEM: Short Time-series Expression Mine STEM
TAO1: Target of AvrB operation 1
Tex: Thioredoxins
TTSS: Type III secretion system
VW: Verticillum wilt
ZED1: HOPZ-ACTIVARED RESISTANCE 1
